# “Three‐in‐one” Analysis of Proteinuria for Disease Diagnosis through Multifunctional Nanoparticles and Machine Learning

**DOI:** 10.1002/advs.202410751

**Published:** 2025-01-15

**Authors:** Yidan Wang, Jiazhu Sun, Jiuhong Yi, Ruijie Fu, Ben Liu, Yunlei Xianyu

**Affiliations:** ^1^ Department of Clinical Laboratory of Sir Run Run Shaw Hospital College of Biosystems Engineering and Food Science Zhejiang University School of Medicine Hangzhou 310058 P. R. China; ^2^ Key Laboratory of Precision Medicine in Diagnosis and Monitoring Research of Zhejiang Province Sir Run Run Shaw Hospital Hangzhou 310016 P. R. China; ^3^ Department of Urology The First Affiliated Hospital Zhejiang University School of Medicine Hangzhou 310058 P. R. China

**Keywords:** machine learning, multifunctional nanoparticles, noninvasive diagnosis, sensor array, urine analysis

## Abstract

Urinalysis is one of the predominant tools for clinical testing owing to the abundant composition, sufficient volume, and non‐invasive acquisition of urine. As a critical component of routine urinalysis, urine protein testing measures the levels and types of proteins, enabling the early diagnosis of diseases. Traditional methods require three separate steps including strip testing, protein/creatinine ratio measurement, and electrophoresis respectively to achieve qualitative, quantitative, and classification analyses of proteins in urine with long time and cumbersome operations. Herein, this work demonstrates a “three‐in‐one” protocol to analyze the urine composition by combining multifunctional nanoparticles with machine learning. This work constructs a sensor array to analyze proteinuria by employing nanoparticles with unique optical properties, outstanding catalytic activity, diverse composition, and tunable structure as probes. Different proteins interacted with nanoprobes differently and are classified by this sensor array based on their physicochemical heterogeneities. With the aid of machine learning, the urine composition is precisely detected for the diagnosis of bladder cancer. This protocol enables quantification and classification of 5 proteinuria in 10 min without any tedious pretreatment, showing proimise for the comprehensive analysis of body fluid for early disease diagnosis.

## Graphical abstract

A sensor array‐based “three‐in‐one” protocol is developed for proteinuria analysis through multifunctional nanoparticles and machine learning. Different proteins can be classified by machine learning based on their distinct physicochemical interactions with nanoparticles possessing optical property and catalytic activity, offering a promising approach for body fluid analysis and disease diagnosis.

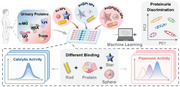

## 1 Introduction

As an information‐rich fluid, urine contains multiple components like glucose, urea, protein, and inorganic salts that can reflect the health status of individuals in the point‐of‐care test.^[^ ^1^, ^2^, ^3^ ^]^ Compared with blood, urine contains potential early‐stage disease biomarkers that are not subject to homeostasis regulation, thus better reflecting the physiological changes in patients.^[^ ^4^ ^]^ Meanwhile, urine, with its low complexity and good stability, can be collected continuously and non‐invasively, making it safer and less costly than blood collection.^[^ ^5^, ^6^ ^]^ Therefore, routine urinalysis is crucial in health monitoring, providing a basis for assessing metabolic and excretory functions, and enabling early disease diagnosis. Urine protein screening is a vital part of routine urine testing because the protein composition in urine is closely associated with the health conditions of individuals.^[^ ^7^ ^]^ Proteinuria, characterized by abnormal levels of urine protein, is recognized as a warning signal for individuals to monitor their health. Its presence relates to chronic kidney diseases such as glomerulonephritis and tubulointerstitial nephritis, and can result from diabetic nephropathy and multiple myeloma.^[^ ^8^, ^9^ ^]^ Therefore, protein classification and proteinuria analysis are essential for identifying pathogenesis and screening diseases in clinical diagnosis and prognosis assessments.^[^ ^10^ ^]^

Traditional urine‐routine tests remain the predominant tools for diagnosing and monitoring proteinuria in clinical settings. In traditional urinalysis, strip testing is initially used as a qualitative test to confirm the presence of proteinuria, followed by 24‐h urine protein quantification or protein/creatinine ratio measurement to quantify protein concentration in the urine. Subsequently, electrophoresis is performed to classify the protein in urine, providing a preliminary diagnosis of the pathogenesis. Traditional methods involve three separate tests to achieve qualitative, quantitative, and classification analyses of urine protein, requiring tedious pretreatment, cumbersome steps, expensive equipments, and highly skilled analysts,^[^ ^11^ ^]^ which significantly limit the broad applications in proteinuria‐related disease screening. Besides, traditional methods generally rely on “lock‐and‐key” recognition strategies that merely enable one‐component target detection while there are multiple analytes in urine samples.^[^ ^12^, ^13^, ^14^ ^]^ Therefore, it is crucial to develop a “three‐in‐one” analytical protocol to enable multiplexed detection of proteinuria in a simple and rapid way without complicated instruments.^[^ ^15^ ^]^

With significant progress in nanotechnology, nanoparticles with tunable optical properties and catalytic activities have emerged as effective tools in biosensing,^[^ ^16^, ^17^, ^18^ ^]^ bacteria resistance,^[^ ^19^, ^20^ ^]^ cancer therapeutics, and drug delivery.^[^ ^21^, ^22^ ^]^ Gold nanoparticles (Au NPs), with unique optical properties dependent on their shapes and plasmonic properties, have been widely utilized as signal probes.^[^ ^23^, ^24^, ^25^, ^26^ ^]^ Based on the catalytic activity of platinum nanoparticles, Au@Pt NPs display outstanding catalytic activities that can be modulated by their intrinsic physicochemical properties.^[^ ^27^, ^28^ ^]^ For proteinuria, it may contain plasma proteins filtered through glomerulus and proteins secreted by kidney and urinary tract, such as tubular proteins (lysozyme, α‐microglobulin (α‐MG)), albumin, and glomerular proteins (transferrin, immunoglobulin (IgG)). Proteins exhibit specific binding affinities with different types of nanoparticles, determined by their molecular weight, isoelectric point, redox capacity, and structural conformation.^[^ ^29^, ^30^ ^]^ Nanoparticles with versatile optical properties and catalytic activities can serve as colorimetric probes for readout. Consequently, differences in electrostatic interactions and steric hindrance among proteins can be amplified and converted into colorimetric signals.^[^ ^31^ ^]^

**Scheme 1:**
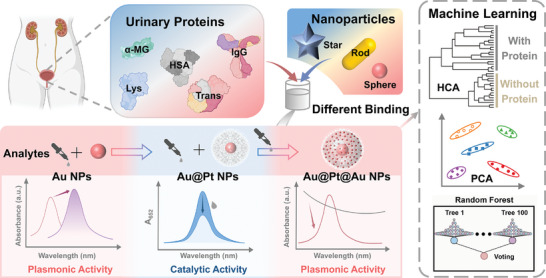
Schematic illustration of the working principle of the “three‐in‐one” multifunctional nanoparticles‐based sensor array for urine analysis.

Herein, we present a “three‐in‐one” multifunctional nanoparticles‐based sensor array for proteinuria analysis through machine learning. Electrostatic interactions and steric hindrance play crucial roles in the differential binding between nanoparticles and urine proteins. By manipulating the charge, size, and shape of nanoparticles, they can preferentially bind with proteins. Owing to the physicochemical heterogeneities of proteins, they can interact with nanoparticles and generate diverse colorimetric signals based on the plasmonic properties and catalytic activities of nanoparticles.^[^ ^32^ ^]^ The unique optical properties of AuNPs are influenced by the size, while the catalytic activities of Au@Pt NPs are modulated by proteins based on surface interactions. Meanwhile, proteins with different properties can modulate the surface growth of gold on Au@Pt NPs, forming Au@Pt@Au NPs for signal readout. In combination with machine learning that can analyze spectroscopic signals and reduce dimensionality, the fingerprint properties of proteins are extracted for accurate classification and reliable diagnosis of bladder cancer (**Scheme** **1**). Five disease‐related proteins can be accurately quantified and classified within 10 min using this “three‐in‐one” analytical protocol without any pretreatment. This work outlines a rapid and convenient strategy for urine analysis, providing guidelines for comprehensive analysis of body fluids for disease diagnosis.

## 2 Results and Discussion

### 2.1 Synthesis and Characterization of Nanoparticles

**Figure 1:**
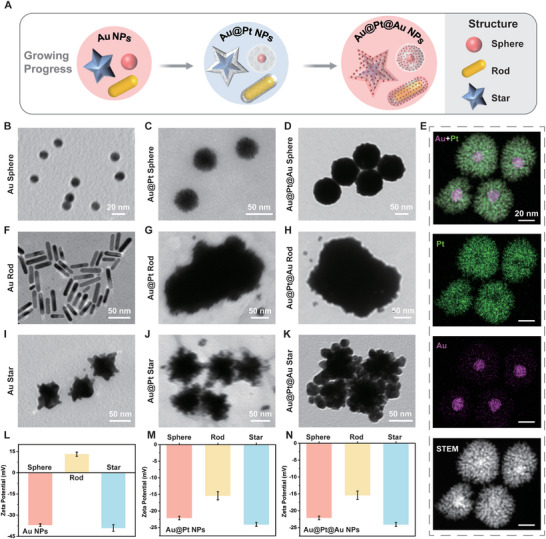
Structural and compositional characterizations of nanoparticles. A) Schematic illustration of the growth of different nanoparticles. B–D) TEM images of Au sphere, Au@Pt sphere, and Au@Pt@Au sphere. E) STEM image and EDS mapping images of Au@Pt spheres. F–H) TEM images of Au rod, Au@Pt rod, and Au@Pt@Au rod. I–K) TEM images of Au star, Au@Pt star, and Au@Pt@Au star. L) Zeta potential of Au sphere, Au rod, and Au star. M) Zeta potential of Au@Pt sphere, Au@Pt rod, and Au@Pt star. N) Zeta potential of Au@Pt@Au sphere, Au@Pt@Au rod, and Au@Pt@Au star.

In this work, we synthesized multifunctional nanoparticles including nanospheres, nanorods, and nanostars through chemical reduction and in situ growth (**Figure** **1**A). Citrate‐capped Au spheres with a diameter of 18 nm exhibited good dispersion and uniformity by transmission electron microscopy (TEM) (Figure 1B). Using an in situ growth method, we synthesized Au@Pt sphere by employing Au sphere as seeds.^[^ ^33^ ^]^ TEM images showed that the surface of Au@Pt spheres changed from smooth to dendritic after depositing platinum on Au spheres (Figure 1C). Furthermore, we coated Au shells on Au@Pt spheres via the chemical reduction between HAuCl_4_ and ascorbic acid. The transition from dendritic to smooth surface confirmed the formation of Au@Pt@Au spheres (Figure 1D). Dynamic light scattering (DLS) analysis confirmed the in situ growth of gold nanoparticles. The hydrodynamic size of Au spheres increased from 20 nm to 81 nm after platinum deposition and further increased to 133 nm after the growth of Au shells (Figure S1A, Supporting Information). To determine the elemental compositions of nanostructures, we used energy dispersive spectroscopy (EDS) mapping to characterize the core‐shell structure. After platinum deposition, Au@Pt spheres presented a core‐shell structure with Au (purple) as the core and Pt (green) as the shell (Figure 1E). Au@Pt@Au spheres exhibited a sandwich‐like structure after the growth of Au shell. The core and the shell were Au (purple) and the interior among them was Pt (green), indicating the formation of Au shells on Au@Pt NPs (Figure S2, Supporting Information).

We further prepared Au nanorods using cetyltrimethylammonium bromide as a surfactant and Ag⁺ to direct anisotropic growth, enabling the selective deposition of gold atoms on seeds. TEM images confirmed the rod‐shape of Au rods, with 48.35 ± 1.54 nm in length and 9.54 ± 0.92 nm in width (Figure 1F). Following this, we deposited Pt on Au rods and TEM images confirmed the anisotropic dendritic structure (Figure 1G). We prepared Au@Pt@Au rods through further coating of Au shell (Figure 1H). The hydrodynamic size peaks of the initial Au rods shifted after in situ growth, indicating the successive platinum and gold deposition (Figure S1B, Supporting Information). Additionally, we synthesized Au nanostars with a branched structure featuring multiple protruding tips (Figure 1I). We further prepared Au@Pt stars and Au@Pt@Au stars through platinum deposition and subsequent gold coating (Figure 1J,K). DLS confirmed the structural change from two hydrodynamic size distributions to one hydrodynamic size after surface growth (Figure S1C, Supporting Information).

The surface charge of nanoparticles plays an essential role in determining their interaction with proteins. We measured the zeta potential, showing that Au spheres and Au stars exhibited negative charges with zeta potentials of −37 and −39.1 mV, respectively. Au rods exhibited a zeta potential of 13.1 mV (Figure 1L). Following platinum deposition, Au@Pt NPs exhibited negative zeta potentials of −10.1, −7.7, and −5.9 mV in spheres, rods, and stars, respectively (Figure 1M). Au@Pt@Au NPs showed negative zeta potentials of −22.1, −15.4, and −12.3 mV in spheres, rods, and stars, respectively (Figure 1N).

### 2.2 Plasmonic and Catalytic Properties of Nanoparticles

**Figure 2:**
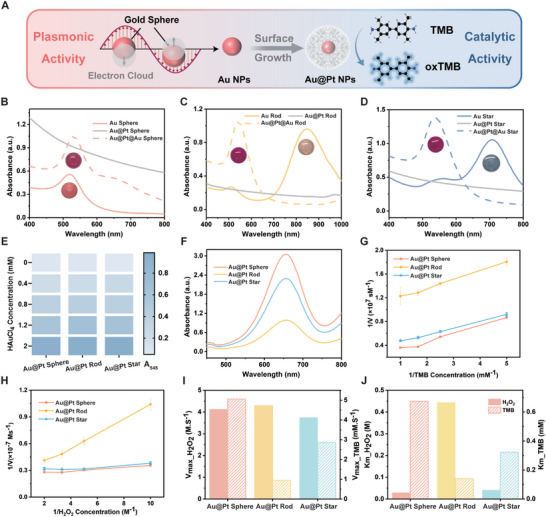
The plasmonic properties and catalytic abilities of nanoparticles. A) The conversion between the plasmonic activity and the catalytic property of nanoparticles. B) UV–vis spectra of Au spheres, Au@Pt spheres, and Au@Pt@Au spheres. C) UV–vis spectra of Au rods, Au@Pt rods, and Au@Pt@Au rods. D) UV–vis spectra of Au stars, Au@Pt stars, and Au@Pt@Au stars. E) Effect of Au content on the plasmonic signal of Au@Pt@Au NPs. F) Typical absorbance spectra of the peroxidation of TMB in the presence of Au@Pt NPs. Kinetic assays of Au@Pt NPs by double‐reciprocal plots with G) TMB and H) H_2_O_2_. I) V_max_ and J) K_m_ value of Au@Pt NPs toward TMB and H_2_O_2._

The plasmonic properties and catalytic activities of nanoparticles play crucial roles in the sensing mechanism of this strategy.^[^ ^34^ ^]^ Citrate‐capped Au spheres exhibited a wine‐red color with a characteristic absorption peak at 520 nm. When coated with platinum, this absorption peak disappeared while the formed Au@Pt spheres exhibited catalytic activity (**Figure** **2**A). After the growth of Au shells, Au@Pt@Au spheres displayed enhanced SPR absorption at 530 nm (Figure 2B). Au rods had a longitudinal peak at 875 nm and a transverse peak at 510 nm. Platinum deposition decreased their plasmonic property but enhanced their catalytic activity. Further growth of Au shells on Au@Pt rods enhanced the SPR absorption peak at 545 nm (Figure 2C). Au stars had an intense longitudinal band at 710 nm and a weaker transverse band at 560 nm. Similarly, platinum deposition resulted in an enhanced catalytic activity and further gold coating increased the SPR absorption at 540 nm (Figure 2D). We introduced HAuCl_4_ as the precursor, and the increased absorption of Au@Pt@Au NPs at 545 nm validated that their plasmonic properties originated from the generation of Au shells (Figure 2E).

Subsequently, we investigated the catalytic activities of Au@Pt NPs utilizing 3,3′,5,5′‐tetramethylbenzidine (TMB), a typical substrate of horseradish peroxidase. The absorption peak at 652 nm (A_652_) was measured to verify the oxidation of TMB, producing blue‐colored oxTMB. Due to the decisive role of platinum in the catalytic activity, Au@Pt NPs exhibited the highest catalytic activity in the spherical shape, performing slightly worse in star, and showing the least activity in rod (Figure 2F). To study the catalytic efficiency of different Au@Pt NPs, we quantified their steady‐state kinetic parameters for the oxidation of TMB with H_2_O_2_. We obtained the Michaelis‐Menten curves by plotting initial reaction velocities against substrate concentrations. The catalytic oxidation of Au@Pt NPs followed typical Michaelis‐Menten behavior toward both TMB and H_2_O_2_ (Figure S3, Supporting Information). We obtained parallel slope lines by converting these curves to double‐reciprocal plots and calculated their kinetic parameters (Figure 2G,H). The V_max_ and K_m_ values among Au@Pt NPs are different, and the V_max_ value of Au@Pt spheres toward TMB is significantly higher than the others, while the V_max_ values toward H_2_O_2_ are similar. Therefore, Au@Pt NPs demonstrated different peroxidase‐like activities beneficial for sensor array applications (Figure 2I,J). The stability of Au NPs and Au@Pt NPs was monitored over 7 days, showing no significant changes in their plasmonic activities and catalytic activities, confirming their excellent stability (Figure S4, Supporting Information).

### 2.3 Sensor Array for Discrimination of Urine Contents

**Figure 3:**
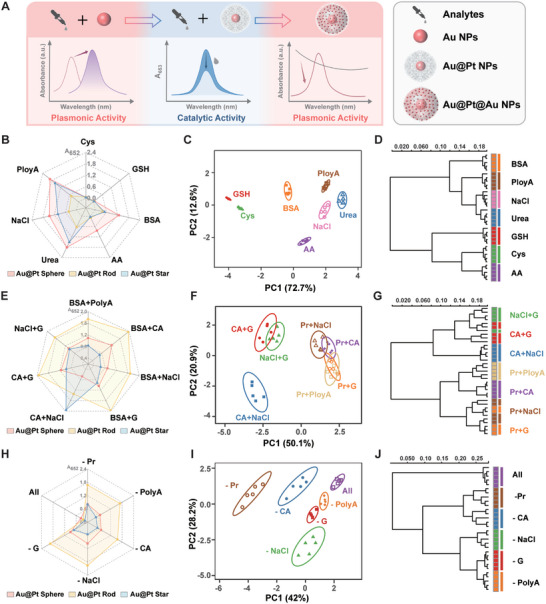
Multifunctional nanoparticles‐based sensor array for analyzing urine components in water. A) Schematic illustration of the effect of bioanalytes on the properties of nanoparticles. B) Radar map derived from catalytic signals (A_652_) for 7 bioanalytes. C) Canonical score plot for two factors of colorimetric response patterns toward 7 bioanalytes obtained from PCA with 95% confidence ellipses (n = 5). D) Cluster tree of 7 bioanalytes by HCA. E) Radar map derived from catalytic signals (A_652_) for 7 dual‐bioanalyte mixtures. F) Canonical score plot toward 7 dual‐bioanalyte mixtures. G) Cluster tree of 7 dual‐bioanalyte mixtures by HCA. H) Radar map derived from catalytic signals (A_652_) for 6 multiple‐bioanalytes mixtures. I) Canonical score plot toward 6 multiple‐bioanalytes mixtures. J) Cluster tree of 6 multiple‐bioanalytes mixtures by HCA. (Acronyms in this figure: BSA represents bull serum albumin, GSH represents glutathione, Cys represents cysteine, AA represents ascorbic acid, CA represents citric acid, Pr represents protein, and G represents glucose).

Urine consists of water, protein, glucose, inorganic salts, and other small molecules, which can be monitored from a macroscopic perspective rather than a specific “lock‐and‐key” recognition model. We fabricated a multifunctional nanoparticles‐based sensor array to monitor urine contents (**Figure** **3**A). Differing in surface charges and shapes, Au NPs could bind with heterogeneous bioanalytes, producing unique localized surface plasmonic resonance and corresponding color changes. We used UV–vis spectrophotometry to record plasmonic signals and calculated the absorbance ratios (A_635_/A_520_, A_875_/A_520_, and A_710_/A_520_) as benchmarks to quantify the dispersion status of Au spheres, rods, and stars respectively, where A_520_, A_635_, A_875,_ and A_710_ were the absorbance at 520, 635, 875 and 710 nm (Figure S5A–C, Supporting Information). The resultant absorbance ratios demonstrated that bioanalytes influenced the dispersion of nanoparticles, generating diverse colorimetric signals (Figure S5D–F, Supporting Information). Similarly, Au@Pt NPs exhibited different affinities with bioanalytes causing diverse steric hindrances, altering their interactions with TMB and H_2_O_2_. By measuring the A_652_ value of oxTMB catalyzed by Au@Pt NPs, we evaluated the effect of the analytes on the interaction between nanoparticles and substrates. The radar maps of A_652_ proved that bioanalytes affected the interaction between Au@Pt NPs and substrates, producing different colorimetric responses due to their physiochemical properties (Figure 3B). Additionally, different bioanalytes affected the growth of Au shells on the surface of Au@Pt NPs, leading to distinct color changes. The formation of Au@Pt@Au NPs exhibited characteristic absorbance peaks around 545 nm (A_545_) that could be used to quantify the growth of Au shells (Figure S6, Supporting Information).

As a highly effective tool, machine learning can process valuable information using computational algorithms that has great potential in analyzing spectroscopic signals.^[^ ^35^, ^36^ ^]^ Dimensionality reduction techniques, such as principal component analysis (PCA) can reduce redundant, noisy, or otherwise uninformative features from extensive datasets. We conducted five replicates for each bioanalyte and constructed a training dataset (9 sensing elements × 7 bioanalytes × 5 replicates). We generated a two‐dimensional (2D) plot utilizing two primary discrimination factors. The canonical colorimetric response plot exhibited a unique fingerprint for each bioanalyte, distinctly separating 7 bioanalytes (Figure 3C). We utilized hierarchical cluster analysis (HCA) to generate a cluster tree that effectively differentiated seven bioanalytics into separated clusters. The results indicated that sodium chloride and urea exhibited similar response patterns, whereas ascorbic acid demonstrated distinct responses due to its reducing ability (Figure 3D). Beyond single bioanalyte discrimination, we further examined the discrimination ability of the multifunctional nanoparticles‐based sensor array for dual‐bioanalyte mixtures. We randomly mixed two bioanalytes and the dual‐bioanalyte mixtures induced diverse colorimetric responses. Different bioanalyte pairs caused varying dispersion of Au rods and Au spheres (Figure S7, Supporting Information). The varying A_652_ values in the radar map demonstrated that bioanalyte pairs influenced the interaction between Au@Pt NPs and substrates (Figure 3E). Different pairs also affected the growth of Au shells on Au@Pt spheres, while weakly influencing the surface growth on Au@Pt rods and Au@Pt stars (Figure S8, Supporting Information). Intriguingly, the dual‐bioanalyte mixtures were classified into three groups in the converted PCA pattern, with all protein‐containing mixtures clustered into one group (Figure 3F). HCA revealed a cluster of protein‐containing mixtures, highlighting that proteins significantly influenced the response patterns in the sensor array (Figure 3G).

Additionally, we employed this sensor array to identify multiple bioanalytes, each formed by randomly removing one bioanalyte from a set of seven mixtures. Multiple‐bioanalyte mixtures affected Au NPs and exhibited different UV–vis spectra (Figure S9, Supporting Information). The seven‐bioanalyte mixture significantly weakened the catalytic abilities of Au@Pt spheres (Figure 3H). Multiple‐bioanalyte mixtures minimally impacted the formation of Au@Pt@Au spheres, except that the protein‐free group (‐Pr), which showed a unique spectrum and highest plasmonic signal (Figure S10, Supporting Information). These signals were separated into six distinct clusters without overlap in the PCA pattern (Figure 3I). HCA revealed that mixtures containing all components showed distinct response patterns, emphasizing the significant contribution of each component to the overall response (Figure 3J). These results implied that this sensor array maintained discrimination ability in multiple‐bioanalyte mixtures, demonstrating great potential for analyzing complex biological samples.

### 2.4 Discrimination of Components in Artificial Urine

**Figure 4:**
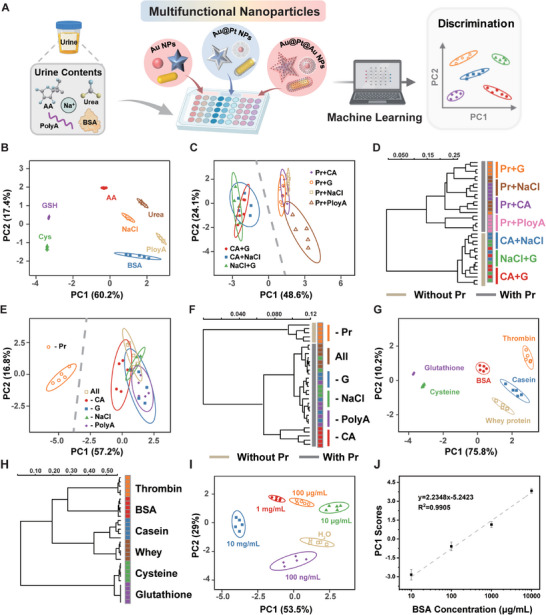
Multifunctional nanoparticles‐based sensor array for discriminating components and proteins in artificial urine. A) Schematic illustration of the mechanism of sensing urine contents. B) PCA score plots for the identification of 7 bioanalytes in artificial urine. C) PCA score plots for the identification of 7 dual‐component mixtures in artificial urine. D) Cluster tree of 7 dual‐component mixtures in artificial urine by HCA. E) PCA score plots for the identification of 6 multiple‐component mixtures in artificial urine. F) Cluster tree of 6 multiple‐component mixtures in artificial urine by HCA. G) PCA score plots for the type identification of 6 representative proteins in artificial urine. H) Cluster tree of 6 representative proteins in artificial urine by HCA. I) PCA score plots for the discrimination of BSA of different concentrations. J) The linear relationship between the first discriminant factor and the concentration of BSA. (Acronyms in this figure: CA represents citric acid, Pr represents protein, Whey represents Whey proteins, and G represents glucose).

We further employed this sensor assay to discriminate single bioanalyte in artificial urine. It showed a variety of unique colorimetric responses, demonstrating its feasibility in discriminating different bioanalytes (**Figure** **4**A). Glutathione, bovine serum albumin (BSA), and cysteine were the most influential for Au NPs (Figure S11, Supporting Information). Bioanalytes significantly affected the catalytic ability of Au@Pt NPs in artificial urine (Figure S12, Supporting Information). Additionally, these analytes influenced the surface growth of Au shells on Au@Pt NPs, leading to distinct A_545_ values and UV–vis spectra (Figure S13, Supporting Information). The 2D canonical score plot, using the first two foremost discrimination factors, effectively differentiated bioanalytes based on their unique fingerprints (Figure 4B). We further evaluated its applicability with complex mixtures by randomly mixing two types of bioanalytes in artificial urine. These mixtures primarily affected the A_520_ value in Au spheres, with minimal impact on that in Au rods and Au stars (Figure S14, Supporting Information). Notably, only protein‐containing groups exhibited unique effects on the catalytic performance of Au@Pt NPs (Figure S15, Supporting Information) and the surface growth of Au shells (Figure S16, Supporting Information). The 2D canonical score plot discriminated mixtures based on the absence or presence of proteins (Figure 4C). HCA revealed a cluster of protein‐containing mixtures, highlighting the significant influence of proteins on the response patterns (Figure 4D). For multiple‐component mixtures, only the group lacking protein exhibited noticeable differences in both the catalytic and plasmonic signals compared to other groups (Figures S17–S19, Supporting Information). Consequently, only mixtures without proteins were separated in the 2D canonical score plot, whereas other groups showed significant overlaps (Figure 4E). HCA revealed that protein‐containing mixtures clustered together, reinforcing the effectiveness of this sensor array for protein analysis (Figure 4F).

We further employed this sensor array to identify different proteins and peptides. The distinct physiochemical properties of proteins and peptides contributed to both the plasmonic signals of Au NPs and the catalytic activities of Au@Pt NPs (Figures S20 and S21, Supporting Information). They also significantly influenced the surface growth of Au shells, leading to the formation of Au@Pt@Au NPs with distinct absorption spectra for classification of proteins and peptides. Owing to sulfhydryl groups, cysteine and glutathione inhibited the growth of Au shells resulting in spectra without any absorption peaks (Figure S22, Supporting Information). We generated a 2D plot using two principle discrimination factors, and the canonical colorimetric response plot enabled separation of different proteins in the transformed PCA patterns (Figure 4G). HCA indicated that thrombin and BSA clustered together due to their similar properties. Casein and whey formed a cluster as dairy proteins, while cysteine and glutathione grouped together as sulfur‐containing compounds (Figure 4H). These results demonstrated that this sensor array effectively classified six proteins. To validate its quantification performance, we used gradient concentrations of BSA as the model protein. Higher concentrations of BSA affected the dispersion of Au NPs and the catalytic abilities of Au@Pt NPs (Figure S23, Supporting Information). The signal responses were plotted in 2D canonical score patterns, enabling separation among clusters of different concentrations (Figure 4I). Furthermore, the first discrimination factor demonstrated a linear relationship with BSA concentration ranging from 10 µg mL^−1^ to 10 mg mL^−1^ (Figure 4J).

### 2.5 “Three‐in‐one” Discrimination of Proteins in Artificial Urine

**Figure 5:**
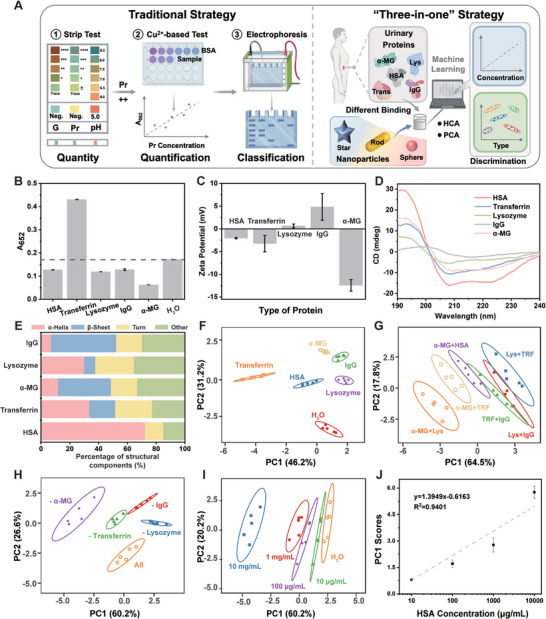
“Three‐in‐one” discrimination of urine proteins. A) Comparison between traditional protocols and the “three‐in‐one” sensor array. B) Redox capacity and C) zeta potentials of urine proteins. D) CD spectra and E) percentage of secondary structures of urine proteins. PCA score plots for the type identification of F) 5 representative proteins, G) 6 dual‐protein mixtures, and H) 5 multiple‐protein mixtures in artificial urine. I) PCA score plots for discriminating HSA of different concentrations. J) The linear relationship between the first discriminant factor and the concentration of HSA. (Acronyms in this figure: Lys represents Lysozyme and TRF represents transferrin).

Urinalysis is a crucial routine test for the early diagnosis and monitoring of diseases where proteinuria can help assess health status based on the concentration and type of urine proteins. Traditional methods for proteinuria detection include strip testing, protein/creatinine ratio measurement, and electrophoresis. Strip testing offers rapid screening via a colorimetric reaction with urinary proteins, but it has limited sensitivity for low‐abundance proteins and is affected by urine composition. Protein/creatinine ratio measurement provides a convenient estimate of protein excretion, though its accuracy may decline in severe kidney impairment. Electrophoresis indentifies specific protein types through separation in an electric field, but it requires skilled technicians. Sequential application of these methods enables qualitative, quantitative, and classification analyses of proteinuria, which is time‐consuming and complicated (**Figure** **5**A). Herein, the developed sensor array enables a “three‐in‐one” discrimination of proteins in a simple and rapid way. We tested human serum albumin (HSA), transferrin, lysozyme, IgG, and α‐MG as representative proteins due to their filtration properties between glomeruli and tubules. HSA, with a molecular weight of 67 kDa, was defined as the cut‐off point to distinguish low molecular‐weight and high molecular‐weight proteins. Proteins with molecular weight lower than 67 kDa such as lysozyme, free light chain, and α‐MG are associated with tubulointerstitial nephritis, while proteins with molecular weight higher than 67 kDa such as transferrin, IgG, and α2‐macroglobulin are associated with glomerulonephritis. Differences in molecular weight lead to variations in sequence length and atom count (Table S1, Supporting Information). We further characterized the redox properties and structural differences of these proteins, contributing to generating distinct plasmonic and catalytic signals. Transferrin displayed outstanding oxidation ability due to iron ions while other proteins had minimal influence (Figure 5B). We investigated the isoelectric point of these proteins by measuring their zeta potentials. Variations in surface charge affected the interaction between proteins and nanoparticles, leading to different dispersion patterns (Figure 5C and Table S1, Supporting Information). We further used the circular dichroism spectrometry to analyze the secondary structure of proteins (Figure 5D,E). Combined with 3D structural data, it showed conformational differences among proteins, particularly the proportions of α‐helix and β‐sheet. Since β‐sheet conformation enhanced protein adsorption onto nanoparticles, IgG and α‐MG exerted a greater impact on the plasmonic and catalytic activities of nanoparticles (Figures 5E and S24, Supporting Information).

We utilized this sensor array to analyze 100 µg mL^−1^ proteins in artificial urine. The spectra of Au spheres shifted significantly in the presence of transferrin, while the intensity of Au stars decreased with the addition of lysozyme or IgG (Figure S25, Supporting Information). Transferrin significantly enhanced whereas other proteins reduced the catalytic activities of Au@Pt spheres (Figure S26, Supporting Information). Surface growth on Au@Pt spheres was significantly inhibited by all proteins, while growth on Au@Pt rods or Au@Pt stars was variably affected, resulting in different spectra and colorimetric signals (Figure S27, Supporting Information). The converted PCA plot demonstrated that these representative proteins were distinctly identified without overlap (Figure 5F). Following this, we employed this sensor array to distinguish dual‐protein mixtures. Different urinary proteins affected the dispersion of Au spheres, resulting in varied spectra (Figure S28, Supporting Information). Lysozyme and transferrin impacted the dispersion of Au spheres, causing a redshift of the characteristic absorption peak and a significant decrease in intensity (Figure S28A, Supporting Information). For Au rods, lysozyme and IgG had minimal effect on their longitudinal peak (Figure S28B, Supporting Information). Pairwise combinations of transferrin, lysozyme, and IgG resulted in higher catalytic signals of Au@Pt NPs (Figure S29, Supporting Information). For the growth of Au shell,α‐MG and lysozyme showed distinctive signals (Figure S30, Supporting Information). We utilized PCA to distinguish dual‐protein mixtures, showing no overlap between the confidence ellipses of different proteins, indicating that dual‐protein mixtures were effectively distinguished by this sensor array (Figure 5G).

Based on dual‐protein discrimination, we further assessed the recognition ability of this sensor array for multiple‐protein mixtures. Different mixtures significantly affected the longitudinal peak of Au rods (Figure S31, Supporting Information). Mixtures lacking α‐MG or IgG exhibited distinct spectra with high absorption intensity in Au spheres, while mixtures without α‐MG or lysozyme were differentiated in Au stars (Figure S31, Supporting Information). The sensor array exhibited the lowest catalytic signal in the presence of five‐protein mixtures (Figure S32, Supporting Information). Simultaneously, the surface growth of Au shells on Au@Pt NPs was affected by these mixtures, leading to varying peak intensities during the formation of Au@Pt@Au NPs (Figure S33, Supporting Information). In PCA plot, these signals formed five independent clusters, accurately identifying these multiple‐protein mixtures (Figure 5H). Besides classifying urinary proteins, qualitative and quantitative tests are also essential for early detection, monitoring, and management of kidney diseases. We assessed the quantitative capability of this sensor array in detecting gradient concentrations of HSA in artificial urine. The colorimetric signals were plotted in 2D canonical score patterns, showing separated clusters corresponding to each concentration (Figure 5I). Additionally, the first discrimination factor was linearly dependent on HSA concentrations from 10 to 10 000 µg mL^−1^ with R^2^ = 0.94 (Figure 5J), avoiding interference from complex matrices compared to conventional bicinchoninic acid assay kits (Figure S34, Supporting Information).

### 2.6 Machine Learning for Diagnosis of Bladder Cancer

**Figure 6:**
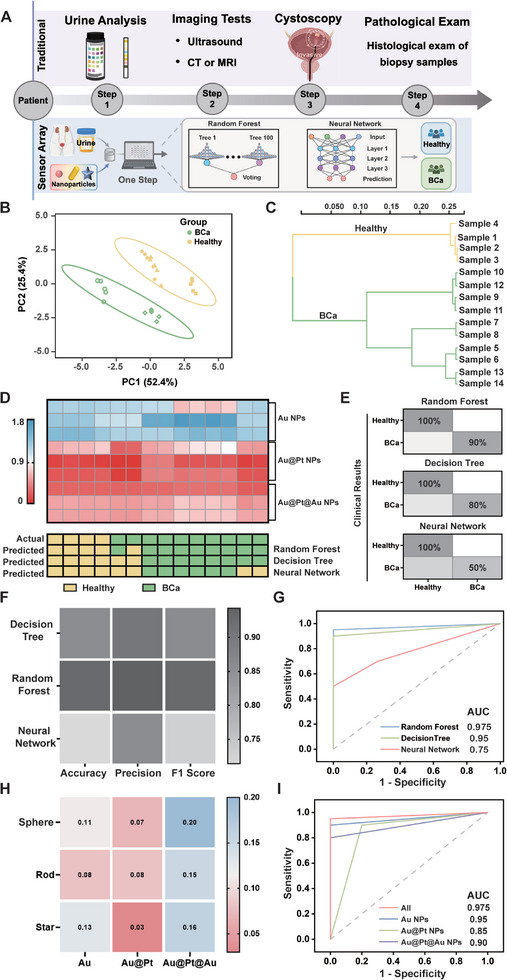
Machine learning for diagnosis of bladder cancer. A) Comparison between clinical diagnosis protocols and machine learning‐assisted sensor array. B) PCA score plots for the identification of BCa patients and healthy individuals. C) Cluster tree of BCa patients and healthy individuals by HCA. D) Application of the trained machine‐learning algorithm to an independent validation cohort. E) Confusion matrix summarizing the BCa identification accuracy by random forest, decision tree, and neural network. F) The accuracy, precision, and F1 score of random forest, decision tree, and neural network. G) ROC curves comparing the success of three algorithms in identifying urine samples. H) Contributions of each probe in the sensor array to the overall accuracy for the identification by random forest. I) ROC curves comparing the success of different probes in identifying urine samples.

Bladder cancer (Bca) is a malignant tumor that poses a serious threat to public health. The presence of biomarkers such as nuclear matrix protein and bladder tumor antigen in urine enables the early detection of Bca, thereby improving cure rates and patient survival rates.^[^ ^37^, ^38^ ^]^ To demonstrate the real‐world application, we employed this sensor array combined with machine learning to distinguish urinary proteins and components of clinical urine samples to diagnose Bca (**Figure** **6**A). For machine learning, we obtained clinical samples from 14 patients and generated a dataset (9 sensing elements × 14 samples × 5 replicates) for BCa diagnosis. We applied PCA to reduce the data dimensionality and generate independent principal components. PCA score plots effectively distinguished between BCa patients and healthy individuals (Figure 6B). We then used HCA to create a cluster tree to indicate separate clusters of BCa patients and healthy individuals (Figure 6C). We applied three machine‐learning algorithms including decision tree, random forest, and neural network to analyze the urine components. All algorithms were trained on 60% of the total data set using supervised learning and the performance was validated by screening BCa from a blinded test set (40% of the total data set) (Figure 6D). Plasmonic and catalytic signals generated from nanoparticles were fed into all algorithms, generating outputs of either healthy or BCa. Notably, it required no pre‐processing that allowed all algorithms to learn from the original urine samples and capture minor characteristics. All algorithms achieved 100% accuracy for “Healthy,” while the accuracies for BCa detection were 90% with random forest, 80% with decision tree, and 50% with neural network (Figure 6E). The random forest algorithm achieved an accuracy of 92.86%, a precision of 94%, and an F1 score of 0.93, demonstrating its outstanding performance (Figure 6F). We further utilized the receiver operating characteristic (ROC) analysis to assess the identification performance of the machine learning models. The random forest model had the largest areas under the ROC curve (AUC), further confirming its superior sensing performance (Figure 6G). Due to the layered architecture optimized via backpropagation to capture complex patterns, neural network is highly effective for intricate data but necessitates a large dataset, rendering it unsuitable for the limited dataset in this study. The limited dataset and its nonlinear characteristics made random forest demonstrate superior performance than the neural network. The random forest model also illustrated the importance of each sensing probe in the decision‐making process. The heat map illustrated the relative importance of each probe in this sensor array, demonstrating that all contributed irreplaceably (Figure 6H). We also generated ROC curves of BCa screening with random forest using different types of nanoparticles. The highest AUC value of 0.975 underscored the significance of the entire sensor array in BCa screening (Figure 6I). This sensor array effectively analyzed untreated urine samples, holding great promise for bladder cancer surveillance.

## 3 Conclusion

In summary, we developed a “three‐in‐one” strategy for analyzing proteinuria in a simple and rapid way based on multifunctional nanoparticles‐based sensor array and machine learning. The sensor array consisted of a series of nanoparticles as probes whose sizes and compositions were modulated by in situ reduction and surface growth. The excellent plasmonic and catalytic properties of nanoparticles enabled discriminating urine components with both the type and concentration of proteins in complex matrixes. Additionally, the practical applications of this sensor array were validated by discriminating the healthy and BCa samples. Taken together, this sensor array exhibited outstanding discrimination ability with excellent accuracy and good reproducibility through machine‐learning‐based identification patterns. It overcame the shortcomings of traditional “lock‐and‐key” patterns and greatly enhanced diagnostic efficiency by achieving the qualitative, quantitative, and classification analyses of urine proteins in a single assay. Encouragingly, this work shows promise in the analysis of body fluids, and the multifunctional nanoparticles‐based sensor array could be broadened to other diagnostic applications that enables non‐invasive detection of diseases without specific biomarkers.

## Conflict of Interest

The authors declare no conflict of interest.

## Supporting information



Supporting Information

## Data Availability

The data that support the findings of this study are available from the corresponding author upon reasonable request.
