# Supplementary material for: “Three‐in‐one” Analysis of Proteinuria for Disease Diagnosis through Multifunctional Nanoparticles and Machine Learning
Source: Adv Sci (Weinh). 2025 Jan 15;12(9):2410751. doi: 10.1002/advs.202410751 (PMC11884592; doi:10.1002/advs.202410751)
Supplement: Supplementary file 1 — Supporting Information [file ADVS-12-2410751-s001.docx]

Supporting Information for

**“Three-in-one” analysis of proteinuria for disease diagnosis through multifunctional nanoparticles and machine learning**

Yidan Wang ^1, 2^, Jiazhu Sun ^3^, Jiuhong Yi ^1, 2^, Ruijie Fu ^1, 2^, Ben Liu ^3^, Yunlei Xianyu ^1, 2 *^

^1^ College of Biosystems Engineering and Food Science, Zhejiang University, Hangzhou 310058, People’s Republic of China

^2^ Key Laboratory of Precision Medicine in Diagnosis and Monitoring Research of Zhejiang Province, Sir Run Run Shaw Hospital, Hangzhou 310016, People’s Republic of China

^3^ Department of Urology, The First Affiliated Hospital, Zhejiang University School of Medicine, Hangzhou 310058, People’s Republic of China

*Corresponding author

E-mail: xianyu19@zju.edu.cn

Content List

MATERIALS AND METHODS S3

SUPPORTING FIGURES 1–34 S7

Figure S1. S7

Figure S2. S8

Figure S3. S9

Figure S4. S10

Figure S5. S11

Figure S6. S12

Figure S7. S13

Figure S8. S14

Figure S9. S15

Figure S10. S16

Figure S11. S17

Figure S12. S18

Figure S13. S19

Figure S14. S20

Figure S15. S21

Figure S16. S22

Figure S17. S23

Figure S18. S24

Figure S19. S25

Figure S20. S26

Figure S21. S27

Figure S22. S28

Figure S23. S29

Figure S24. S30

Figure S25. S31

Figure S26. S32

Figure S27. S33

Figure S28. S34

Figure S29. S35

Figure S30. S36

Figure S31. S37

Figure S32. S38

Figure S33. S39

Figure S34. S40

SUPPORTING TABLES S41

Table S1 S41

**MATERIALS AND METHODS**

**Chemicals and reagents:** Sodium citrate, L-ascorbic acid, 3,3',5,5'-tetramethylbenzidine (TMB), and polyvinyl pyrrolidone (PVP) were purchased from HEOWNS (Shanghai, China). Gold (III) chloride trihydrate (HAuCl_4_·3H_2_O) and hydroxylamine hydrochloride were purchased from Hushi Co., Ltd. (Shanghai, China). Chloroplatinic acid (H_2_PtCl_6_·6H_2_O) were purchased from Sigma-Aldrich (Shanghai, China). Silver nitrate (AgNO_3_), ascorbic acid (AA), Sodium acetate (NaAc) sodium hydroxide (NaOH), and hydrochloric acid (HCl) were purchased from Macklin (Shanghai, China). 4-(2-Hydroxyethyl)-piperazine-1-ethane-sulfonic acid sodium salt (HEPES), cetyltrimethylammonium bromide (CTAB), and sodium borohydride (NaBH_4_) were purchased from Aladdin (Beijing, China). All aqueous solutions were prepared with deionized water.

**Characterization:** The UV–vis spectra were measured with an Infinite 200Pro (TECAN, Switzerland). Dynamic light scattering (DLS) and zeta potential analysis were performed on a Zeta Sizer Nano ZS90 (Malvern Company, England). TEM was performed on a JEOL 2100F with an acceleration voltage of 200 kV.

**Synthesis of Au NPs and Au@Pt NPs:** Au spheres, Au rods, and Au stars were prepared through a seed-mediated growth method. Au NPs were prepared via the citrate-mediated reduction of HAuCl_4_. Typically, HAuCl_4_ (1 mM, 100 mL) was stirred and heated to 120 ℃. Trisodium citrate solution (38.8 mM, 10 mL) was then rapidly introduced and the solution was continuously heated with stirring (1200 rpm). After its color changed from colorless to red, the solution was kept with slow stirring (600 rpm) for 15 min. The solution was cooled at room temperature and filtered by the polyethersulfone membrane.

Au rods were prepared via the surfactant-oriented method with two steps. The Au seed solution was prepared by adding HAuCl_4_ solution (50 mM, 25 μL) to CTAB (100 mM, 4.7 mL), followed by the addition of NaBH_4_ solution (10 mM, 0.3 mL) with vigorous stirring (1000 rpm). The seed solution was obtained after aging for 60 min with yellowish-brown. The growth solution was prepared by mixing CTAB (100 mM, 10 mL), HAuCl_4_ (50 mM, 100 μL), HCl (1 M, 100 μL), AgNO_3_ (10 mM, 120 μL), and AA (100 mM, 80 μL) with stirring. Finally, Au seed solution (120 μL) was added to the growth solution and the obtained mixture was incubated overnight at 27 ℃.

For the synthesis of Au stars, citrate-stabilized Au spheres (750 μL), HEPES (100 mM, 18.75 mL), and water (38.5 mL) were mixed, followed by the addition of hydroxylamine hydrochloride (40 mM, 750 μL) under vigorous stirring (1450 rpm). Then, HAuCl_4_ solution (1 mM, 22.5 mL) was dropwise added to the above solution. The reaction was kept under moderate stirring (700 rpm) for 15 minutes.

Au@Pt NPs were synthesized using a seed-mediated growth method. For the synthesis of Au@Pt NPs, 20% wt PVP (20 μL), ascorbic acid (100 mg/mL, 40 μL), and H_2_PtCl_6_ (100 mM, 40 μL) were added to the prepared Au NPs, and the mixture was incubated and heated at 65 ℃ for 30 min. After that, the solution was cooled down in water and Au@Pt NPs were formed.

**Kinetic Assays:** The peroxidase-like activity of Au@Pt NPs was evaluated as follows. Au@Pt NPs were incubated with NaAc/HAc buffer containing different concentrations of TMB and H_2_O_2_ for 10 min, followed by the measurement of absorbance at 653 nm for 2 min.

**Sensor array for discrimination of analytes:** 20 μL of analytes were mixed with 20 μL of Au NPs for 10 min, and the UV-vis spectra of them were recorded and the absorbance ratios were calculated. 40 μL of analytes were mixed with 40 μL of Au@Pt NPs. The resultant mixtures were incubated for 1 h at 37 ℃. Then, 60 μL of NaAc/HAc buffer containing TMB (0.6 mM) and H_2_O_2_ (100 mM) was added into 40 μL of mixtures to react at room temperature for 2 min, and the absorbance was recorded at 653 nm. Furthermore, 40 μL of the mixture was added to the well that contained HAuCl_4_ (2 mM, 40 μL) and AA (100 mM, 20 μL) to enable the surface growth of Au shells for 10 min. The SPR spectra of Au@Pt@Au NPs were recorded by a UV-vis spectrometer.

**Discrimination of components** **in artificial urine:** The artificial urine was prepared by dissolving 1.20 g urea, 0.016 g uric acid, 0.0944 g trisodium citrate dihydrate, 0.6119 g sodium chloride, 0.4713 g potassium chloride, 0.0462 g magnesium sulfate, 0.03 g calcium chloride, 0.092 g sodium sulfate, and 3.23 g sodium dihydrogen phosphate in 100 mL of water, following established protocols. Dual and multiple-component mixtures were obtained by randomly combining two bioanalytes and removing one bioanalyte from all combinations, respectively. Measurements were conducted using the constructed sensor arrays. The resulting signals were analyzed via PCA and HCA for data classification.

**“Three-in-one” discrimination of proteins in artificial urine:** Different proteins were analyzed through the same procedure for qualitative analysis, quantification, and classification within a single process using the PCA algorithm. The classification of diverse protein types and quantitative analysis of HSA were tested by the PCA algorithm, with PC1 serving as the quantitative standard.

**Machine learning for diagnosis of** **bladder cancer:** The clinical samples were obtained from the First Affiliated Hospital, Zhejiang University School of Medicine to establish a dataset (9 sensing elements × 14 samples × 5 replicates) for BCa diagnosis. Urine samples were directly incubated with nanoparticles to produce colorimetric signals, yielding 6 plasmonic and 3 catalytic signals from the 9 sensing elements per sample. This data formed a two-dimensional matrix for subsequent analysis. A taxonomy file categorized clinical samples, and PCA and HCA were conducted via ClustVis**(**a web tool for visualizing clustering of multivariate data). The classification and accuracy metrics of clinical samples were obtained through random forest, decision tree, and neural network analyses conducted on spssau.com.


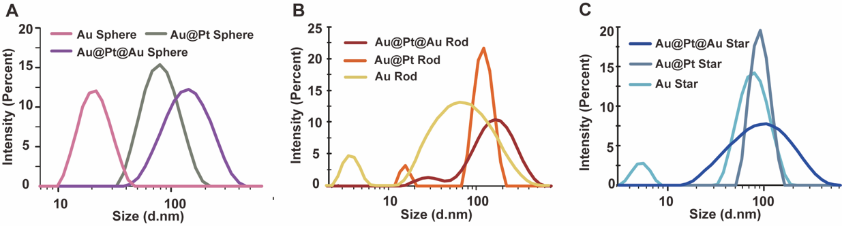


**Figure S1.** DLS analyses of the surface growth on (A) Au sphere, (B) Au rod, and (C) Au star.


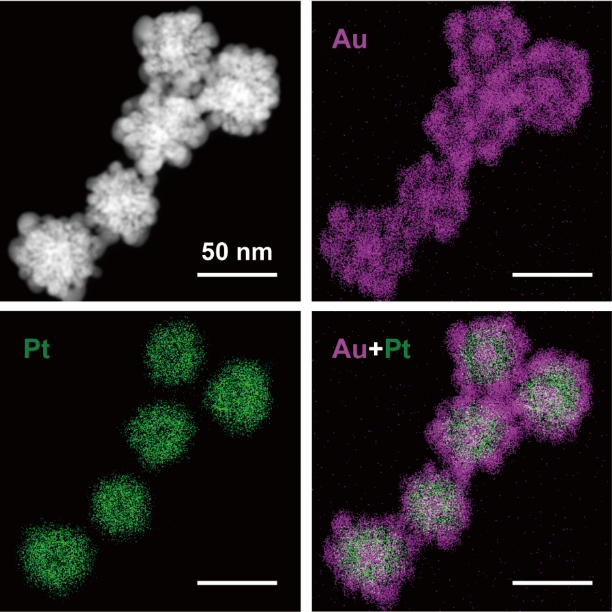


Figure S2. STEM image and EDS mapping images of Au@Pt@Au spheres.


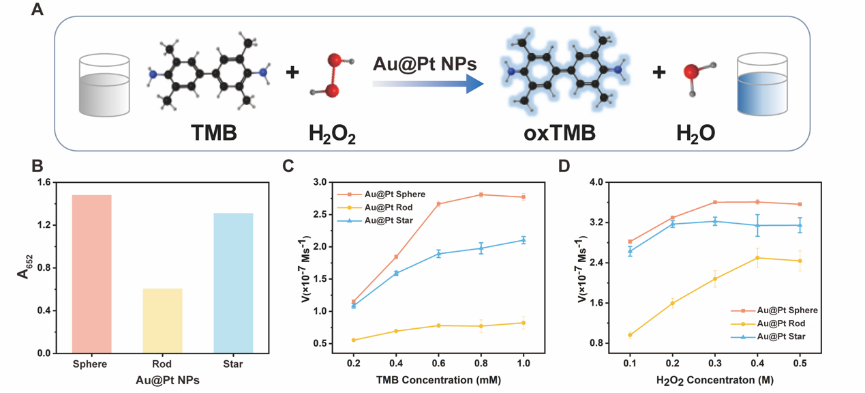


Figure S3. The catalytic activities of Au@Pt NPs. (A) Scheme illustration of the catalytic activity of Au@Pt NPs. (B) Absorbance at 652 nm of TMB oxidation by H_2_O_2_ catalyzed by Au@Pt NPs. (C and D) Steady-state kinetic assays of Au@Pt NPs for the oxidation of TMB by H_2_O_2_. Plot of initial reaction velocity (ν) against (C) TMB and (D) H_2_O_2_ concentration. ν was measured in 0.2 M HAc/NaAc buffer (pH=4.0) at room temperature. In all plots, each point represents the average of three independent measurements.


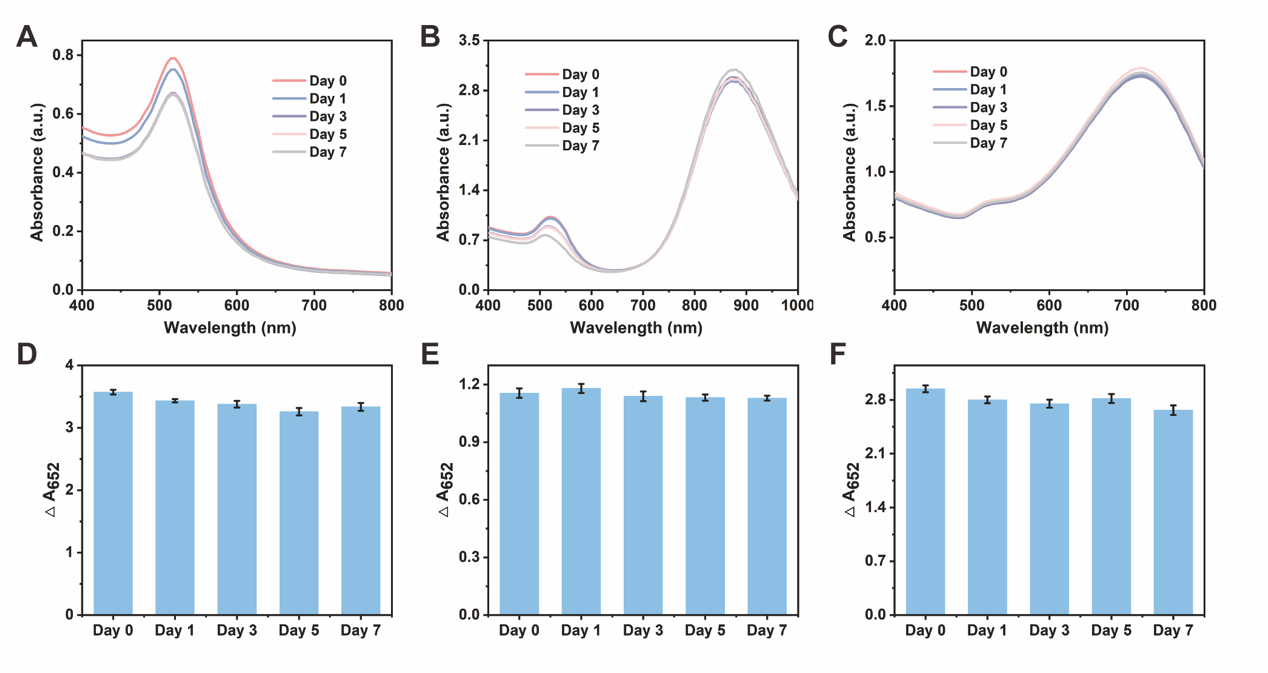


**Figure S4.** The plasmonic activities of (A) Au Sphere, (B) Au Rod, and (C) Au Star over 7 days. The catalytic activities of (D) Au@Pt Sphere, (E) Au@Pt Rod, and (F) Au@Pt Star over 7 days.


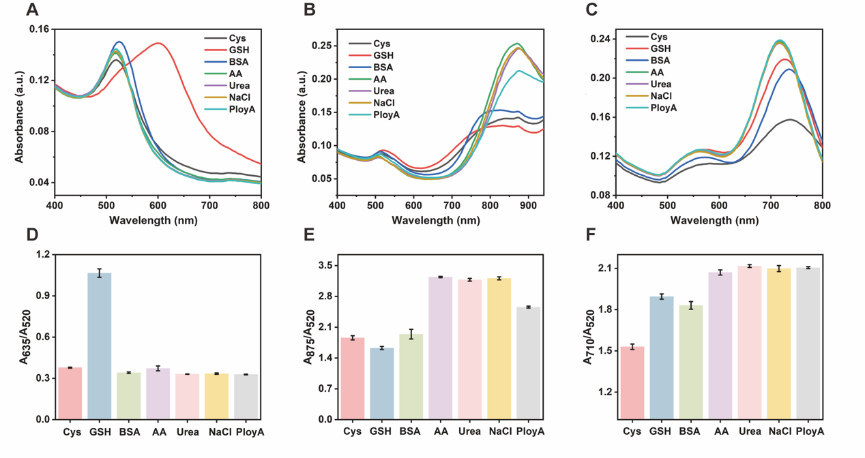


Figure S5. Effect of single bioanalytes on the plasmonic signals of (A) Au sphere, (B) Au rod, and (C) Au star. The calculated absorbance ratios (D) A_635_/A_520_, (E) A_875_/A_520_, and (F) A_710_/A_520_ against 7 bioanalytes. Error bar shows the standard deviation of five independent measurements.


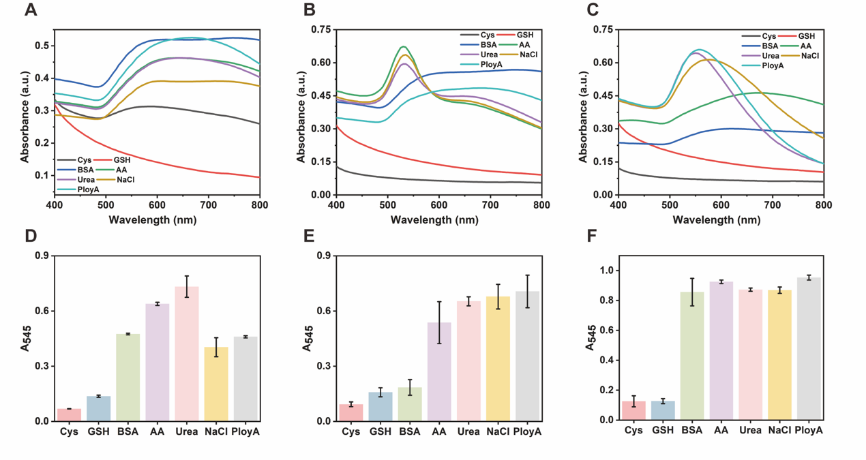


Figure S6. Effect of single bioanalytes on the regrowth progress of Au shells on (A) Au@Pt sphere, (B) Au@Pt rods, and (C) Au@Pt star. The representative plasmonic signals (A_545_) of (D) Au@Pt@Au sphere, (E) Au@Pt@Au rod, and (F) Au@Pt@Au star in response to 7 bioanalytes.


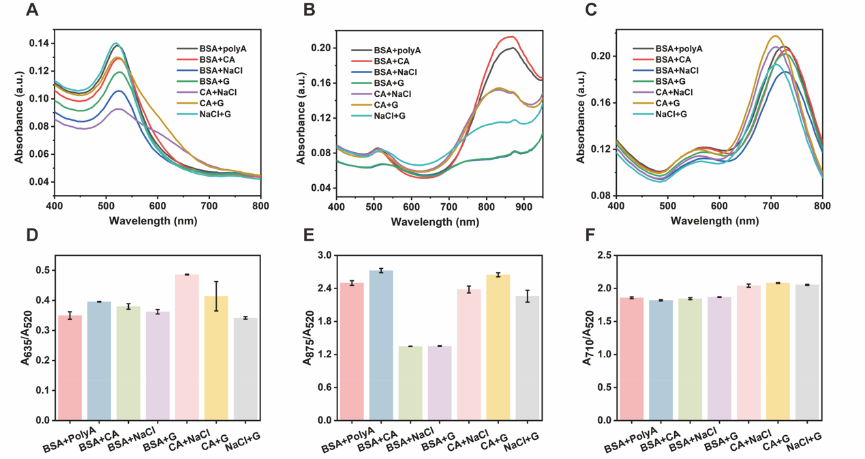


Figure S7. Effect of dual-bioanalyte mixtures on the plasmonic signals of (A) Au sphere, (B) Au rod, and (C) Au star. The calculated absorbance ratios (D) A_635_/A_520_, (E) A_875_/A_520_, and (F) A_710_/A_520_ against dual-bioanalyte mixtures. Error bar shows the standard deviation of five independent measurements.


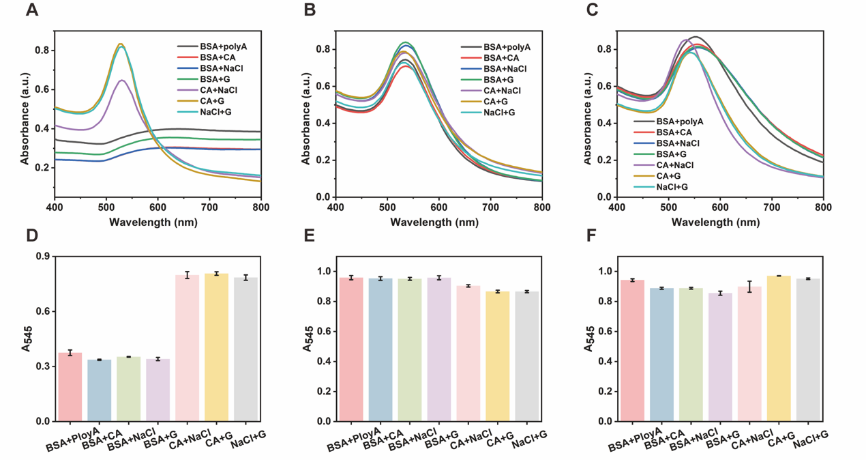


Figure S8. Effect of dual-bioanalyte mixtures on the regrowth progress of Au shells on (A) Au@Pt sphere, (B) Au@Pt rod, and (C) Au@Pt star. The representative plasmonic signals (A_545_) of (D) Au@Pt@Au sphere, (E) Au@Pt@Au rod, and (F) Au@Pt@Au star in response to dual-bioanalyte mixtures. Error bar shows the standard deviation of five independent measurements.


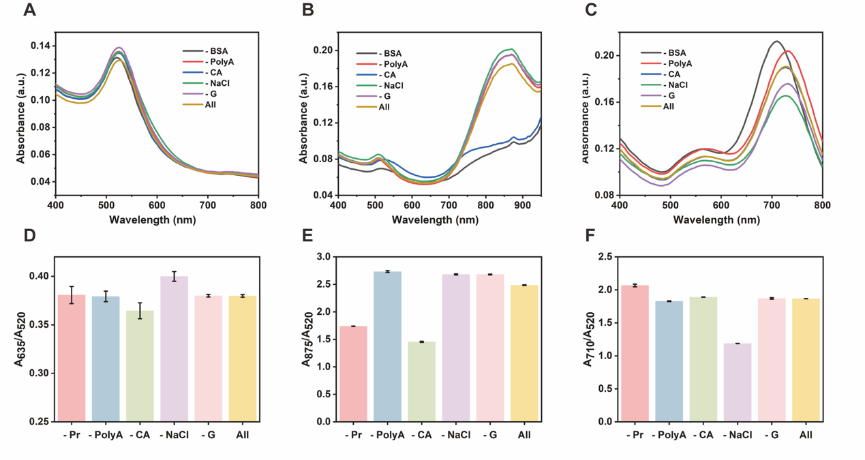


Figure S9. Effect of multiple-bioanalyte mixtures on the plasmonic signals of (A) Au sphere, (B) Au rod, and (C) Au star. The calculated absorbance ratios (D) A_635_/A_520_, (E) A_875_/A_520_, and (F) A_710_/A_520_ against multiple-bioanalyte mixtures. Error bar shows the standard deviation of five independent measurements.


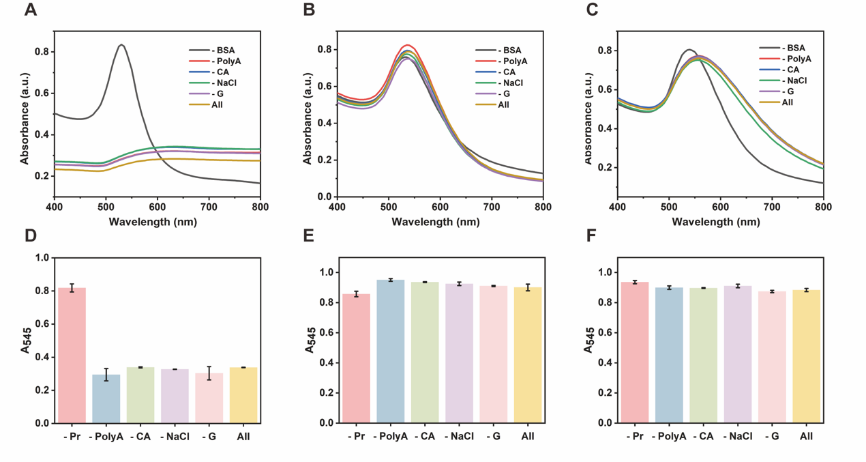


Figure S10. Effect of multiple-bioanalyte mixtures on the regrowth progress of Au shells on (A) Au@Pt sphere, (B) Au@Pt rod, and (C) Au@Pt star. The representative plasmonic signals (A_545_) of (D) Au@Pt@Au sphere, (E) Au@Pt@Au rod, and (F) Au@Pt@Au star in response to multiple-bioanalyte mixtures. Error bar shows the standard deviation of five independent measurements.


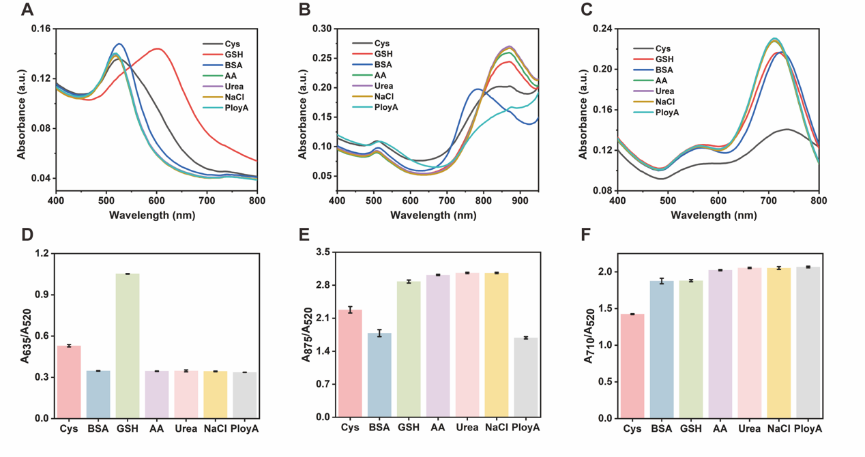


Figure S11. Effect of single bioanalyte on the plasmonic signals of (A) Au sphere, (B) Au rod, and (C) Au star in artificial urine. The calculated absorbance ratios (D) A_635_/A_520_, (E) A_875_/A_520_, and (F) A_710_/A_520_ against single bioanalyte in artificial urine. Error bar shows the standard deviation of five independent measurements.


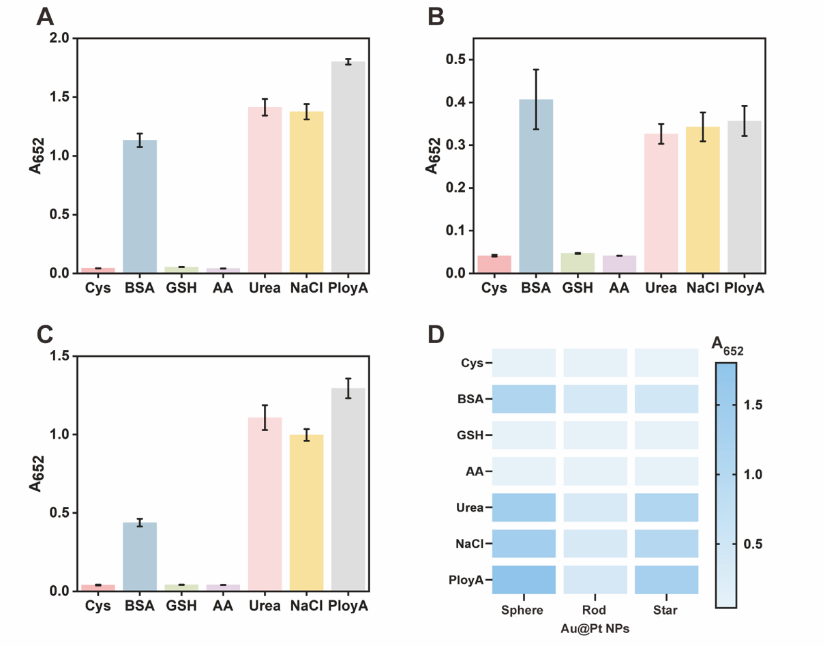


Figure S12. Effect of single bioanalyte on the catalytic signals of (A) Au@Pt sphere, (B) Au@Pt rod, and (C) Au@Pt star in artificial urine. (D) Heat map comparing the catalytic abilities of Au@Pt NPs influenced by seven bioanalytes.


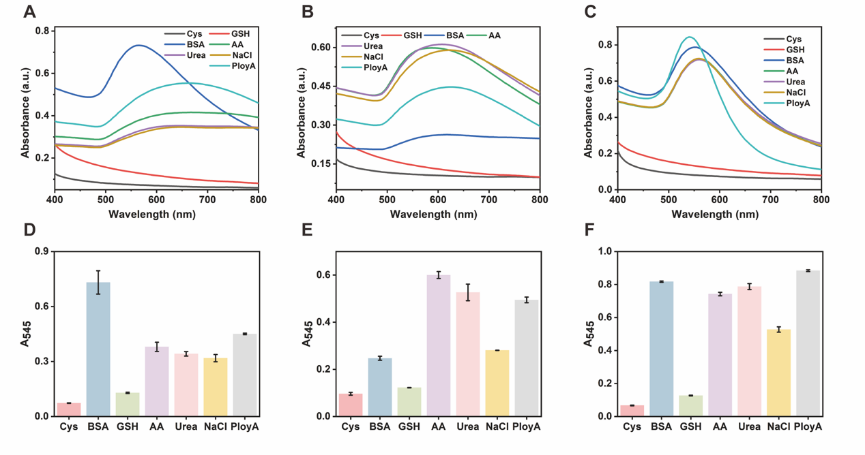


Figure S13. Effect of 7 bioanalyte mixtures on the regrowth progress of Au shells on (A) Au@Pt sphere, (B) Au@Pt rod, and (C) Au@Pt star in artificial urine. The representative plasmonic signals (A_545_) of (D) Au@Pt@Au sphere, (E) Au@Pt@Au rod, and (F) Au@Pt@Au star in response to 7 mixtures in artificial urine. Error bar shows the standard deviation of five independent measurements.


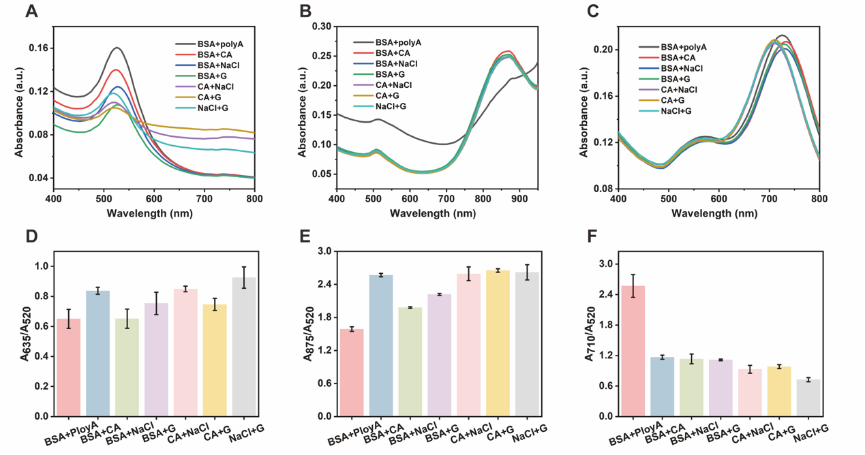


Figure S14. Effect of dual-bioanalyte mixtures on the plasmonic signals of (A) Ausphere, (B) Aurod, and (C) Austar in artificial urine. The calculated absorbance ratios (D) A_635_/A_520_, (E) A_875_/A_520_, and (F) A_710_/A_520_ against dual-bioanalyte mixtures in artificial urine. Error bar shows the standard deviation of five independent measurements.


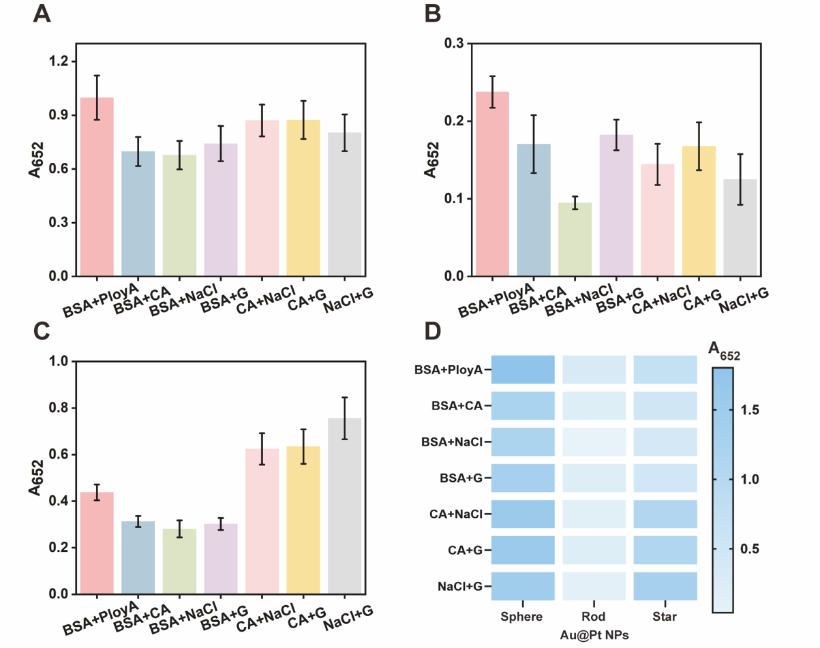


Figure S15. Effect of dual-bioanalyte mixtures on the catalytic signals of (A) Au@Pt sphere, (B) Au@Pt rod, and (C) Au@Pt star in artificial urine. (D) Heat map comparing the catalytic abilities of Au@Pt NPs influenced by dual-bioanalyte mixtures.

**
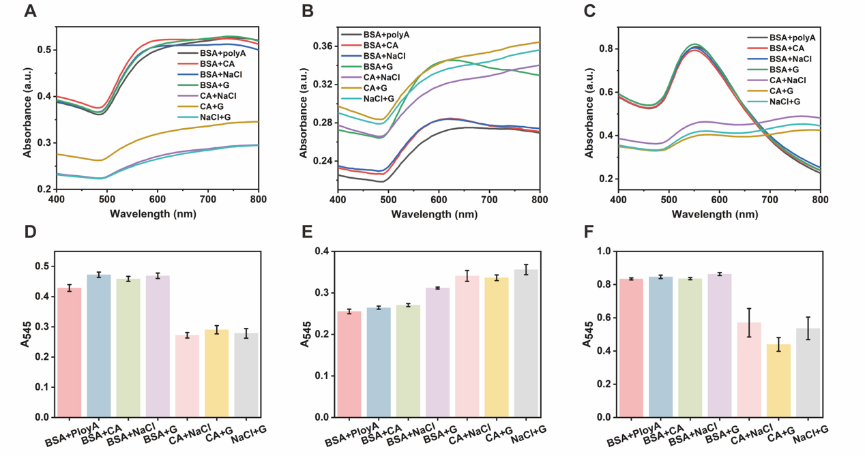
**

Figure S16. Effect of dual-bioanalyte mixtures on the regrowth progress of Au shells on (A) Au@Pt sphere, (B) Au@Pt rod, and (C) Au@Pt star in artificial urine. The representative plasmonic signals (A_545_) of (D) Au@Pt@Au sphere, (E) Au@Pt@Au rod, and (F) Au@Pt@Au star in response to dual-bioanalyte mixtures in artificial urine. Error bar shows the standard deviation of five independent measurements.


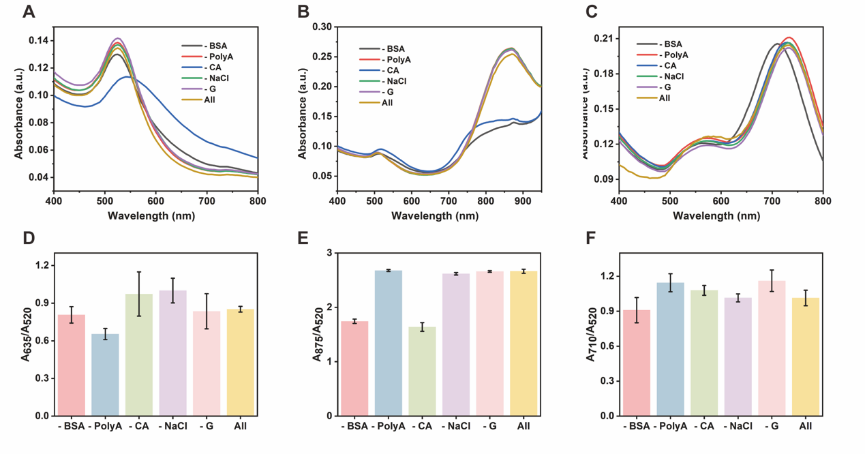


Figure S17. Effect of multiple-bioanalyte mixtures on the plasmonic signals of (A) Ausphere, (B) Aurod, and (C) Austar in artificial urine. The calculated absorbance ratios (D) A_635_/A_520_, (E) A_875_/A_520_, and (F) A_710_/A_520_ against multiple-bioanalyte mixtures in artificial urine. Error bar shows the standard deviation of five independent measurements.


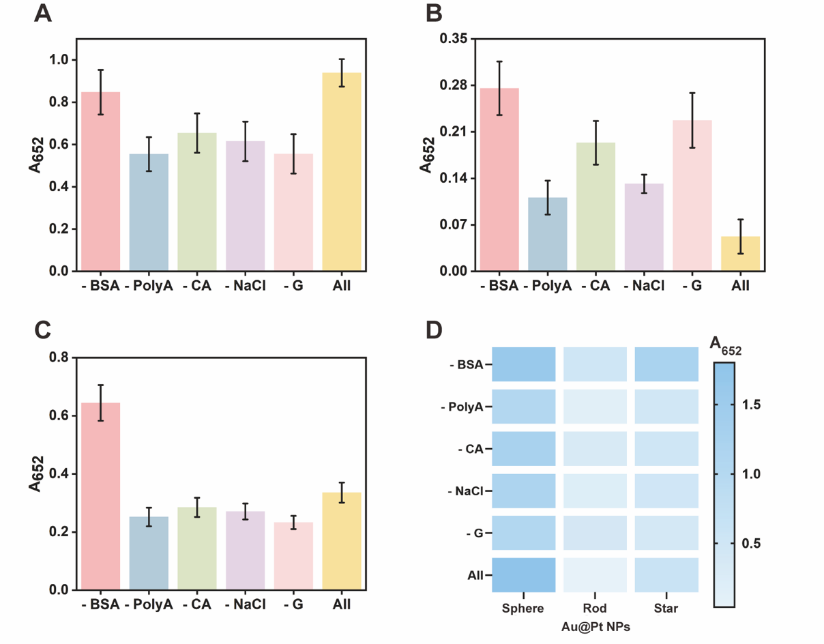


Figure S18. Effect of multiple-bioanalyte mixtures on the catalytic signals of (A) Au@Pt sphere, (B) Au@Pt rod, and (C) Au@Pt star in artificial urine. (D) Heat map comparing the catalytic abilities of Au@Pt NPs influenced by multiple-bioanalyte mixtures.


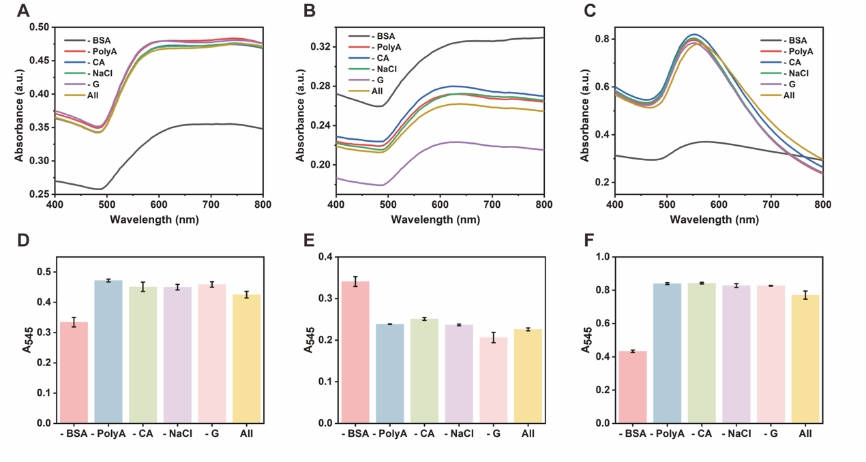


Figure S19. Effect of multiple-bioanalyte mixtures on the regrowth progress of Au shells on (A) Au@Pt sphere, (B) Au@Pt rod, and (C) Au@Pt star in artificial urine. The representative plasmonic signals (A_545_) of (D) Au@Pt@Au sphere, (E) Au@Pt@Au rod, and (F) Au@Pt@Au star in response to multiple-bioanalyte mixtures in artificial urine. Error bar shows the standard deviation of five independent measurements.


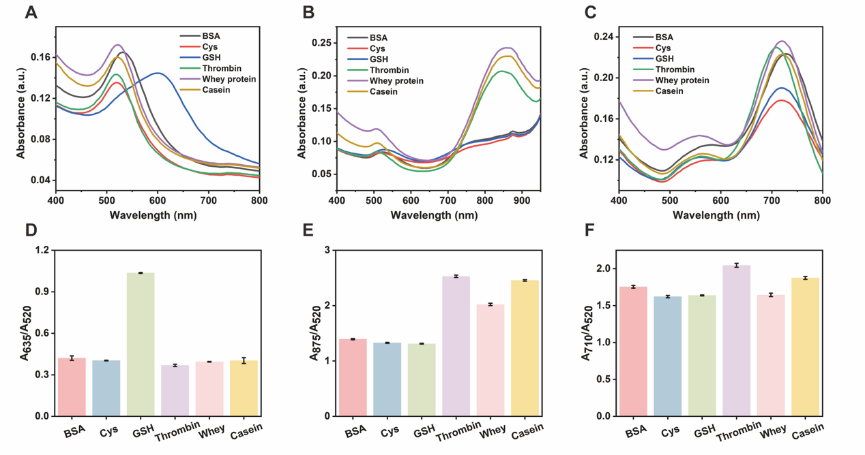


Figure S20. Effect of 6 representative proteins on the plasmonic signals of (A) Au sphere, (B) Au rod, and (C) Au star in artificial urine. The calculated absorbance ratios (D) A_635_/A_520_, (E) A_875_/A_520_, and (F) A_710_/A_520_ against 6 representative proteins in artificial urine. Error bar shows the standard deviation of five independent measurements.


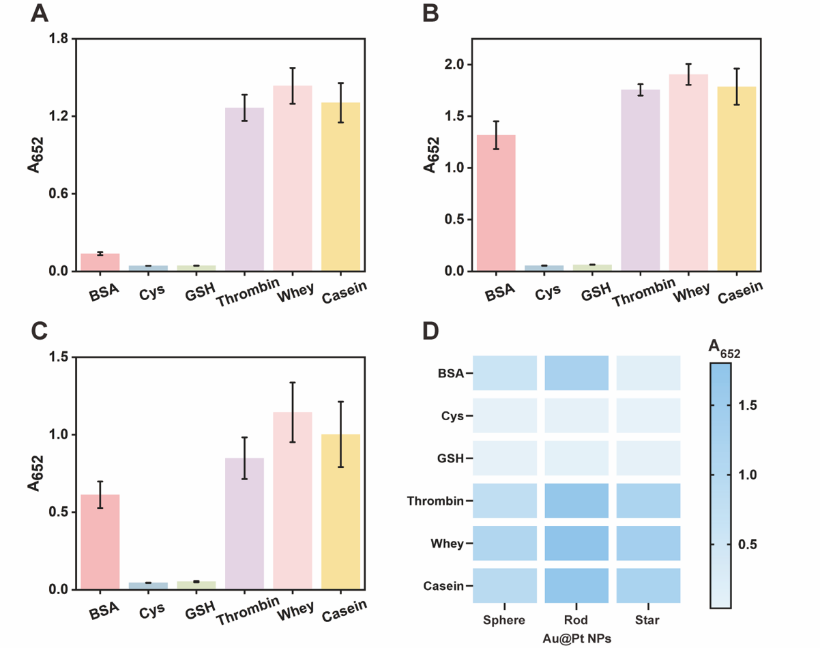


Figure S21. Effect of 6 representative proteins on the catalytic signals of (A) Au@Pt sphere, (B) Au@Pt rod, and (C) Au@Pt star in artificial urine. (D) Heat map comparing the catalytic abilities of Au@Pt NPs influenced by 6 representative proteins.


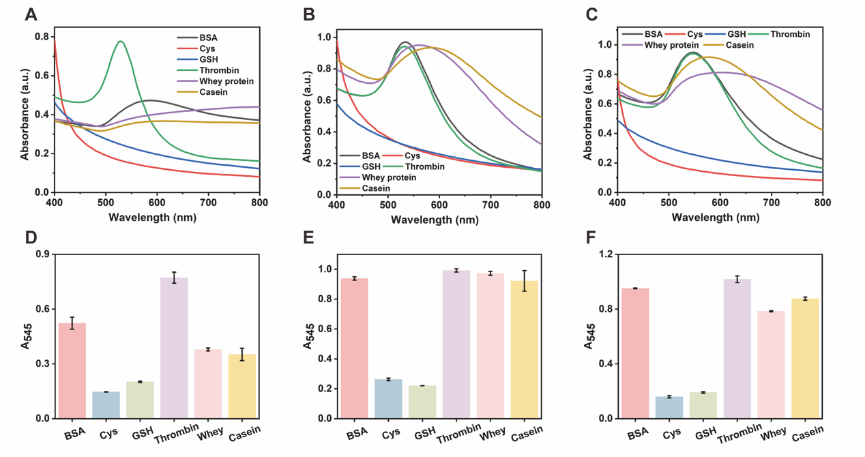


Figure S22. Effect of 6 representative proteins on the regrowth progress of Au shells on (A) Au@Pt sphere, (B) Au@Pt rod, and (C) Au@Pt star in artificial urine. The representative plasmonic signals (A_545_) of (D) Au@Pt@Au sphere, (E) Au@Pt@Au rod, and (F) Au@Pt@Au star in response to 6 representative proteins in artificial urine. Error bar shows the standard deviation of five independent measurements.


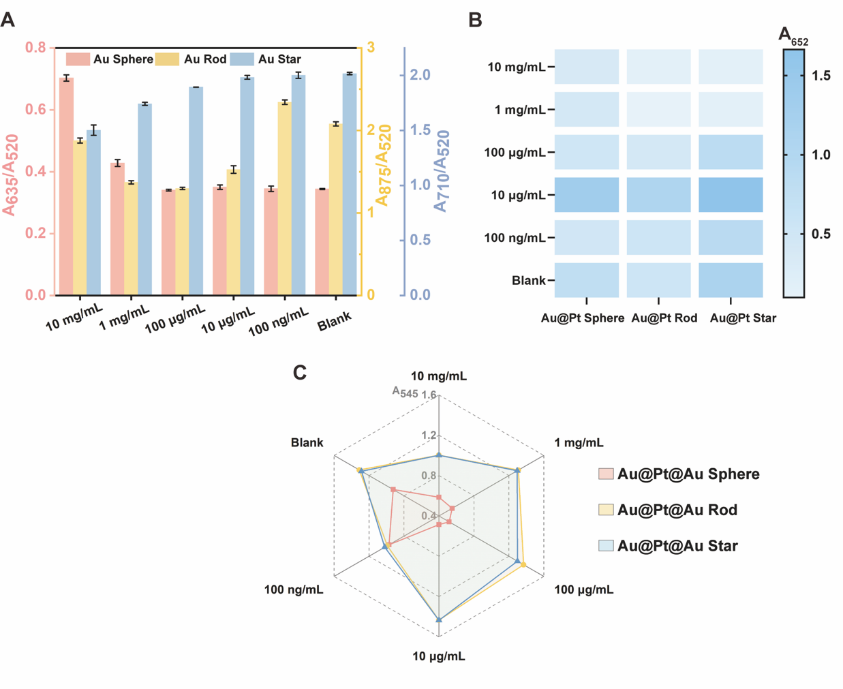


Figure S23. (A) The absorbance ratios (A_635_/A_520_, A_875_/A_520_, A_710_/A_520_) of AuNPs towards BSA against different concentrations in artificial urine. Error bar shows the standard deviation of five independent measurements. (B) Heat map derived from enzyme-like response signals (A_652_) towards BSA against different concentrations in artificial urine. (C) The radar map of A_545_ of Au@Pt@Au NPs in response to BSA against different concentrations in artificial urine.


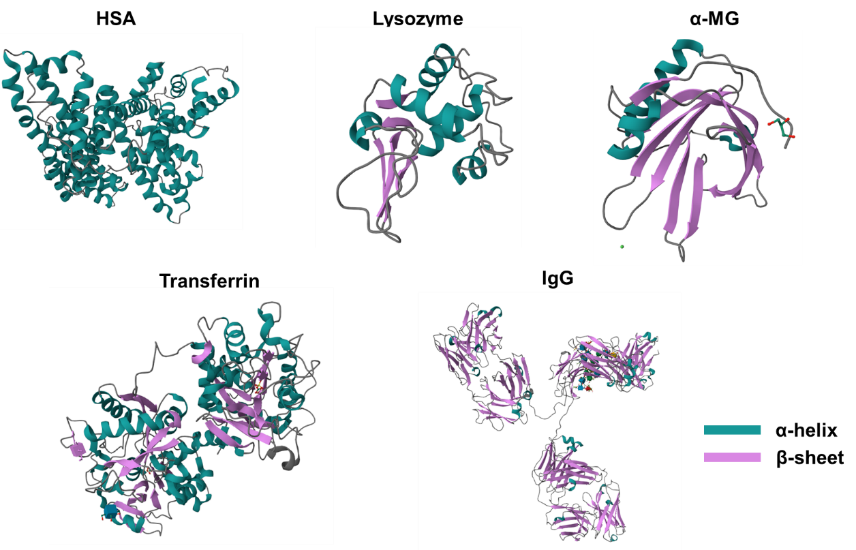


Figure S24. The 3D structure of representative urine proteins.


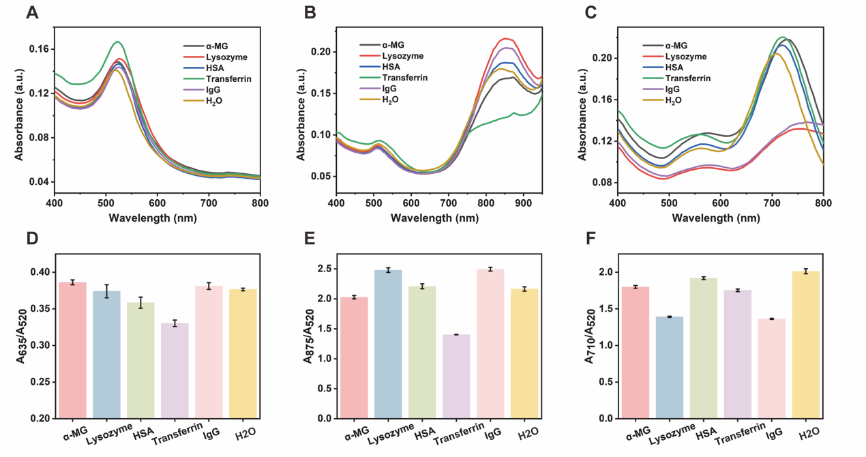


Figure S25. Effect of single urine protein on the plasmonic signals of (A) Au sphere, (B) Au rod, and (C) Au star in artificial urine. The calculated absorbance ratios (D) A_635_/A_520_, (E) A_875_/A_520_, and (F) A_710_/A_520_ against single urine protein in artificial urine. Error bar shows the standard deviation of five independent measurements.


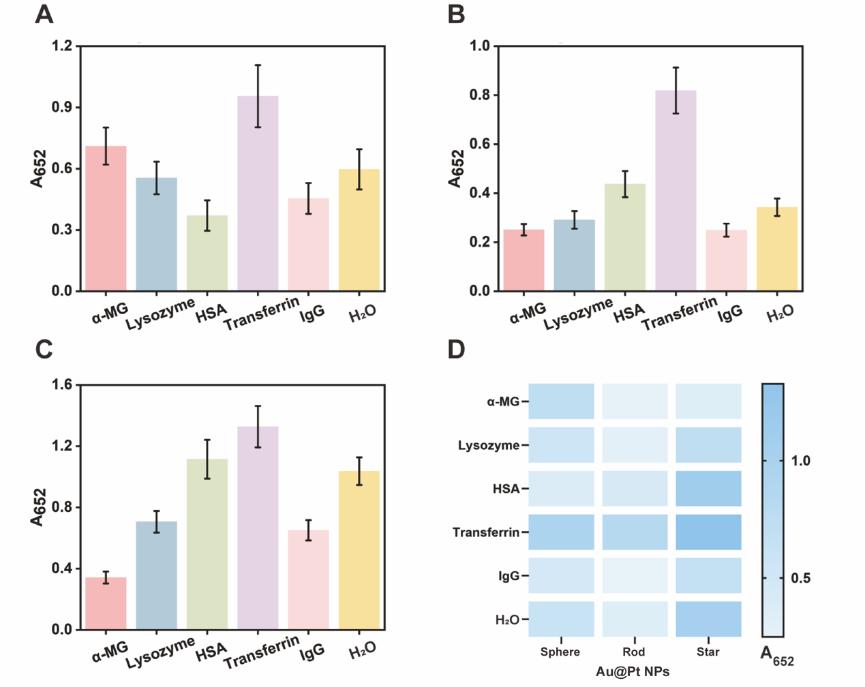


Figure S26. Effect of single urine protein on the catalytic signals of (A) Au@Pt sphere, (B) Au@Pt rod, and (C) Au@Pt star in artificial urine. (D) Heat map comparing the catalytic abilities of Au@Pt NPs influenced by single urine protein. Error bar shows the standard deviation of five independent measurements.


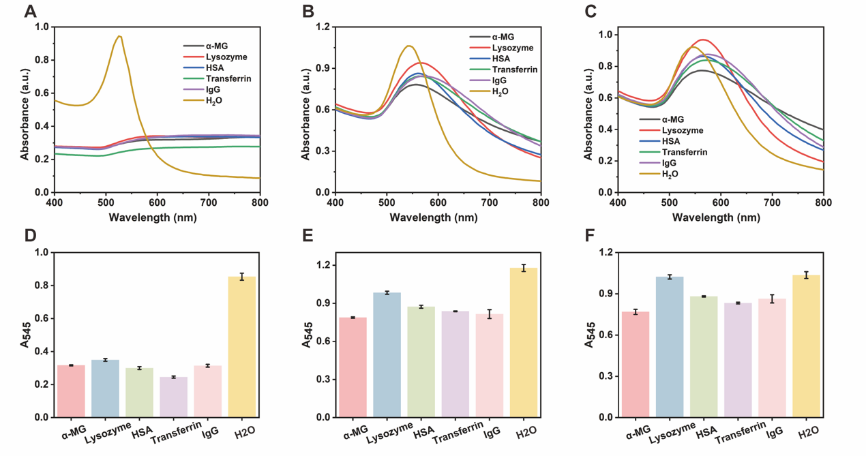


Figure S27. Effect of single urine protein on the regrowth progress of Au shells on (A) Au@Pt sphere, (B) Au@Pt rod, and (C) Au@Pt star in artificial urine. The representative plasmonic signals (A_545_) of (D) Au@Pt@Au sphere, (E) Au@Pt@Au rod, and (F) Au@Pt@Au star in response to single urine protein in artificial urine. Error bar shows the standard deviation of five independent measurements.


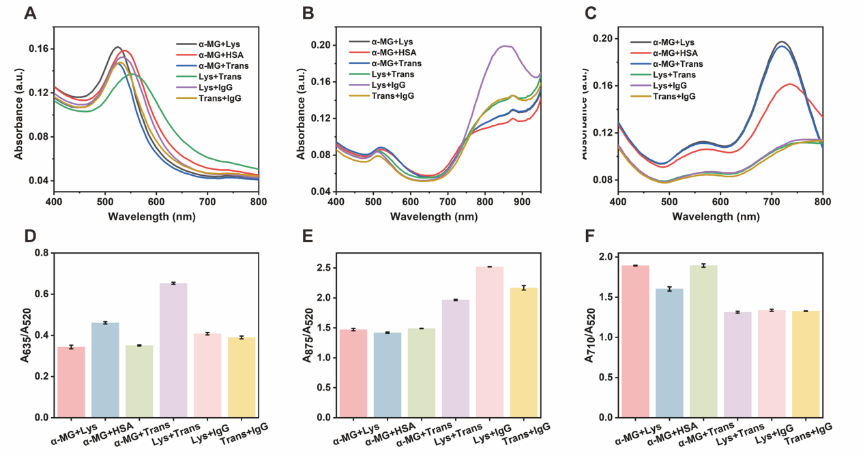


Figure S28. Effect of dual-protein mixtures on the plasmonic signals of (A) Au sphere, (B) Au rod, and (C) Au star in artificial urine. The calculated absorbance ratios (D) A_635_/A_520_, (E) A_875_/A_520_, and (F) A_710_/A_520_ against dual-protein mixtures protein in artificial urine. Error bar shows the standard deviation of five independent measurements.


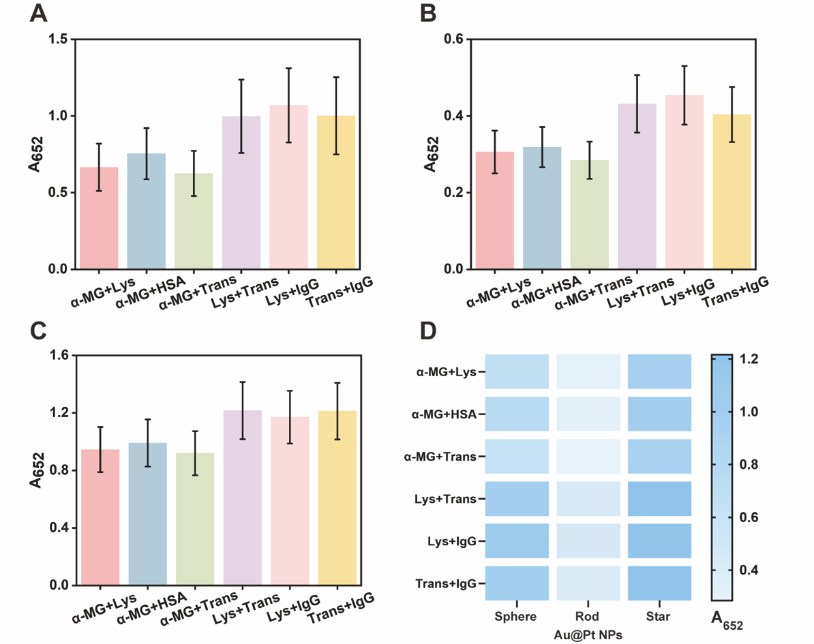


Figure S29. Effect of dual-protein mixtures on the catalytic signals of (A) Au@Pt sphere, (B) Au@Pt rod, and (C) Au@Pt star in artificial urine. (D) Heat map comparing the catalytic abilities of Au@Pt NPs influenced by dual-protein mixtures.


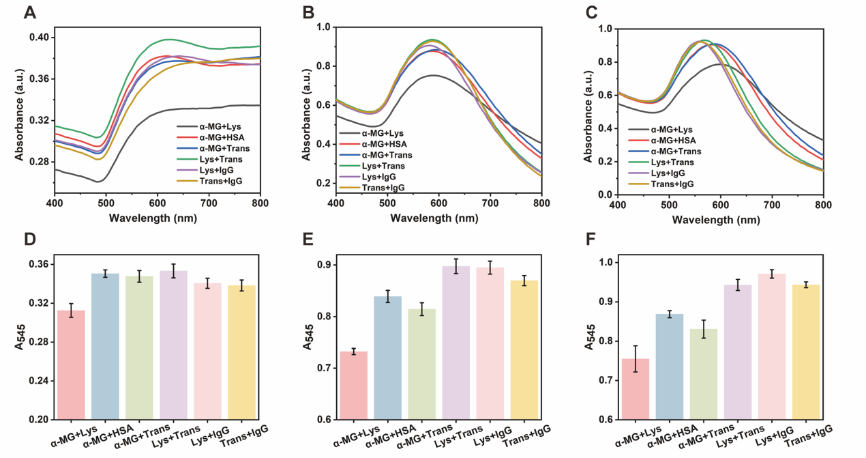


Figure S30. Effect of dual-protein mixtures on the regrowth progress of Au shells on (A) Au@Pt sphere, (B) Au@Pt rod, and (C) Au@Pt star in artificial urine. The representative plasmonic signals (A_545_) of (D) Au@Pt@Au sphere, (E) Au@Pt@Au rod, and (F) Au@Pt@Au star in response to dual-protein mixtures in artificial urine. Error bar shows the standard deviation of five independent measurements.


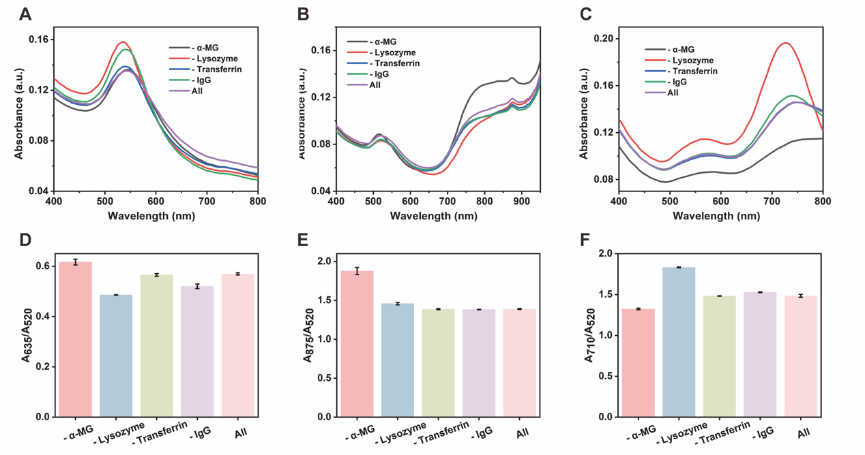


Figure S31. Effect of multiple-protein mixtures on the plasmonic signals of (A) Au sphere, (B) Au rod, and (C) Au star in artificial urine. The calculated absorbance ratios (D) A_635_/A_520_, (E) A_875_/A_520_, and (F) A_710_/A_520_ against multiple-protein mixtures in artificial urine. Error bar shows the standard deviation of five independent measurements.


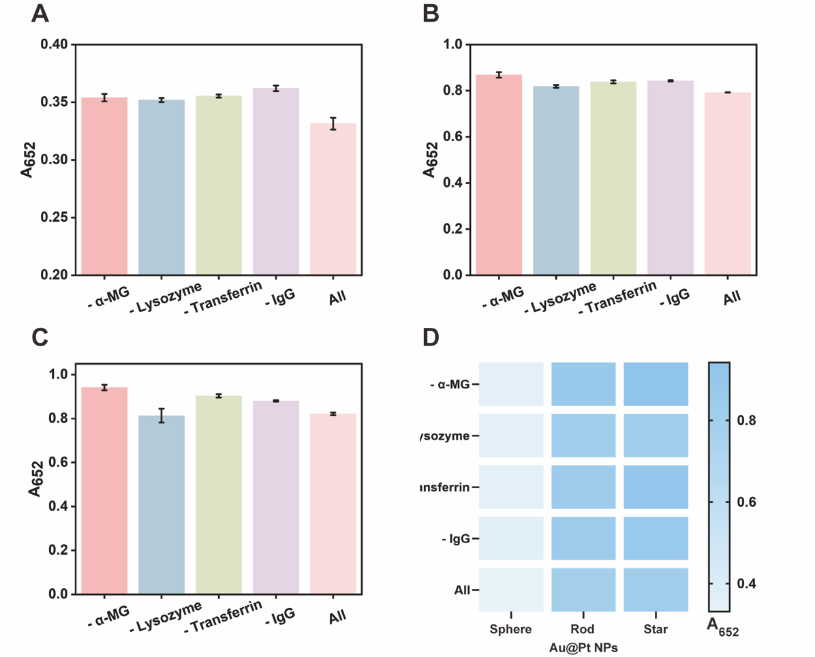


Figure S32. Effect of multiple-protein mixtures on the catalytic signals of (A) Au@Pt sphere, (B) Au@Pt rod, and (C) Au@Pt star in artificial urine. (D) Heat map comparing the catalytic abilities of Au@Pt NPs influenced by multiple-protein mixtures.


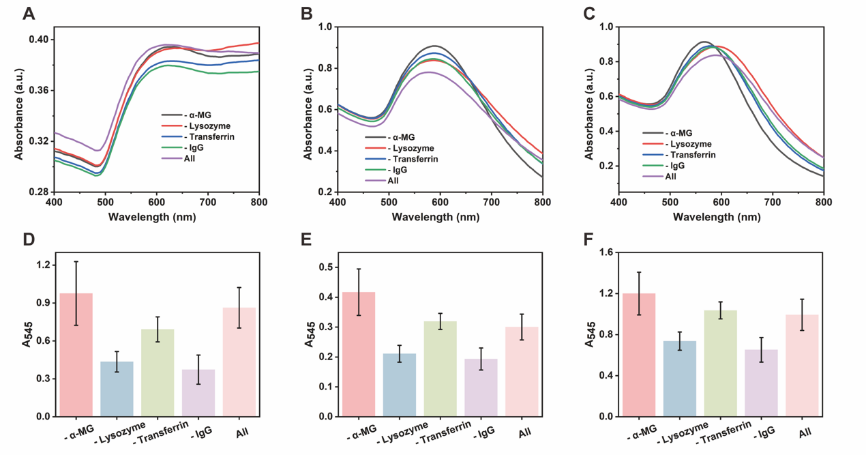


Figure S33. Effect of multiple-protein mixtures on the regrowth progress of Au shells on (A) Au@Pt sphere, (B) Au@Pt rod, and (C) Au@Pt star in artificial urine. The representative plasmonic signals (A_545_) of (D) Au@Pt@Au sphere, (E) Au@Pt@Au rod, and (F) Au@Pt@Au star in response to multiple-protein mixtures in artificial urine. Error bar shows the standard deviation of five independent measurements.


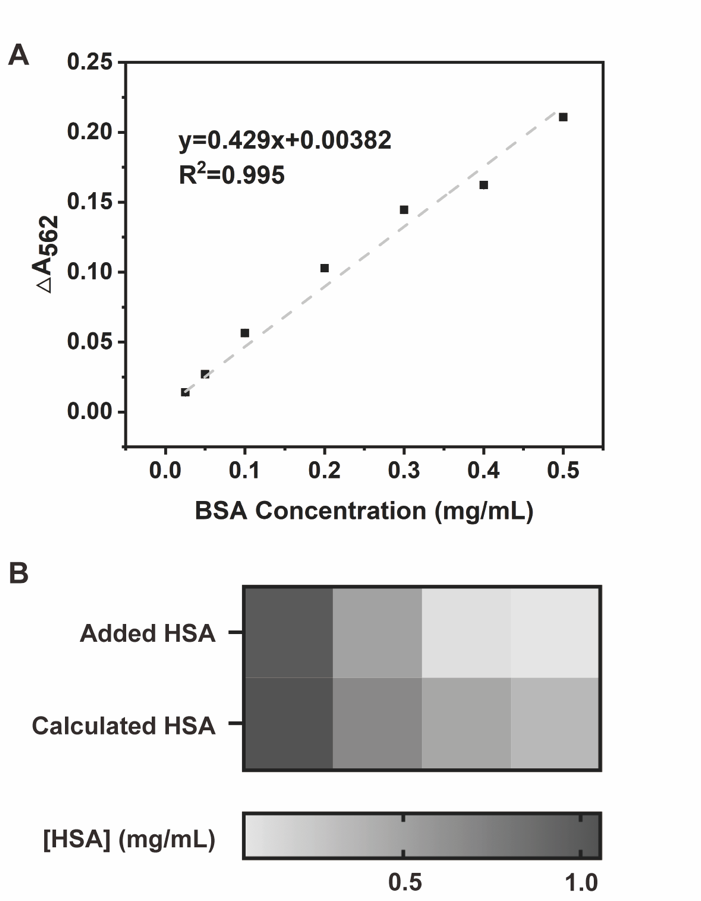


**Figure S34.** (A) Standard curve for BCA assay kit. (B) Results of HSA detection in artificial urine using BCA assay kit.

Table S1. Characterizations of five representative urine proteins

|  | MW  (kDa) | Isoelectric Point | Sequence Length | Atom  Count | Related  Diseases |
| --- | --- | --- | --- | --- | --- |
| HSA | 66.5 | 4.7 | 585 | 9205 | Cut-off Point |
| Transferrin | 79.6 | 5.9 | 679 | 5316 | Glomerular Proteinuria |
| Lysozyme | 14.3 | 11.0 | 130 | 1029 | Tubular Proteinuria |
| IgG | ~150 | 6.1-8.5 | 676 | 10434 | Glomerular Proteinuria |
| α1-MG | 26-31 | 5.4-5.9 | 193 | 1371 | Tubular Proteinuria |

Atom Count: Number of modeled non-hydrogenatoms in the deposited model
